# ProcessGAN: Generating Privacy-Preserving Time-Aware Process Data with Conditional Generative Adversarial Nets

**DOI:** 10.1145/3687464

**Published:** 2024-11-12

**Authors:** KEYI LI, SEN YANG, TRAVIS M. SULLIVAN, RANDALL S. BURD, IVAN MARSIC

**Affiliations:** Electrical and Computer Engineering Department, Rutgers University, New Brunswick, New Jersey, USA; Waymo, Mountain View, CA, USA; Children’s National Hospital, Washington, DC, USA; Children’s National Hospital, Washington, DC, USA; Electrical and Computer Engineering Department, Rutgers University, New Brunswick, NJ, USA

**Keywords:** Synthetic data generation, Process mining, Sequential data, Generative adversarial networks, Data privacy, Time aware

## Abstract

Process data constructed from event logs provides valuable insights into procedural dynamics over time. The confidential information in process data, together with the data’s intricate nature, makes the datasets not sharable and challenging to collect. Consequently, research is limited using process data and analytics in the process mining domain. In this study, we introduced a synthetic process data generation task to address the limitation of sharable process data. We introduced a generative adversarial network, called ProcessGAN, to generate process data with activity sequences and corresponding timestamps. ProcessGAN consists of a transformer-based network as the generator, and a time-aware self-attention network as the discriminator. It can generate privacy-preserving process data from random noise. ProcessGAN considers the duration of the process and time intervals between activities to generate realistic activity sequences with timestamps. We evaluated ProcessGAN on five real-world datasets, two that are public and three collected in medical domains that are private. To evaluate the synthetic data, in addition to statistical metrics, we trained a supervised model to score the synthetic processes. We also used process mining to discover workflows for synthetic medical processes and had domain experts evaluate the clinical applicability of the synthetic workflows. ProcessGAN outperformed the existing generative models in generating complex processes with valid parallel pathways. The synthetic process data generated by ProcessGAN better represented the long-range dependencies between activities, a feature relevant to complicated medical and other processes. The timestamps generated by the ProcessGAN model showed similar distributions with the authentic timestamps. In addition, we trained a transformer-based network to generate synthetic contexts (e.g., patient demographics) that were associated with the synthetic processes. The synthetic contexts generated by our model outperformed the baseline models, with the distributions similar to the authentic contexts. We conclude that ProcessGAN can generate sharable synthetic process data indistinguishable from authentic data. Our source code is available in https://github.com/raaachli/ProcessGAN.

## 1 Introduction

**Process mining (PM)** techniques have been applied to discover knowledge about procedures in different disciplines, including healthcare [32], business [39], and education [4]. Through PM, researchers can understand how a process is performed, identify the bottlenecks and inefficiencies in the process, and improve the process. For example, in healthcare, PM can help optimize patient flow, reduce treatment delays, and enhance the quality of care by identifying inefficiencies or errors in treatment pathways [27, 32]. In business, it can streamline operations, reduce costs, and improve customer satisfaction by analyzing and optimizing workflows [40, 49]. The importance of PM lies in its ability to provide actionable insights from process data, leading to significant improvements in efficiency and effectiveness. Process data in these fields usually contains confidential information, adding a requirement for addressing privacy concerns and obstacles related to data sharing [37]. It is also labor-intensive for humans to collect sufficient process data, considering that it contains varied pathways, timestamps, and contexts and often requires participant consent for use. While data sharing has led to advances in research fields (e.g., ImageNet in computer vision and GLUE in natural language processing) by standardizing research practice and enabling model exploration and benchmark comparisons, public process datasets are still limited compared to other data types such as images, text, and sensor. The lack of data are a barrier to PM studies, which often prevents the replication of research findings. For example, among different healthcare institutions, the scarcity of shared process data can prevent the development and validation of new algorithms for improving patient care pathways. Although data anonymization could be used for data sharing, it does not reduce the risk of user re-identification and loss of data utility, which can hinder meaningful research.

In order to address the data limitation challenge and benefit the PM community, we introduced a task of synthetic process data generation. By generating synthetic data that mimics real-world process data, we aim to provide researchers with the necessary resources to advance the field of PM while maintaining privacy and reducing the burden on data collection. The framework of this task is to train deep generative models (e.g., generative adversarial nets [10]) to learn the underlying data distribution from random noise, and use these models to generate high quality synthetic process data. The synthetic process data with a learnt distribution similar to actual data can be shared without compromising confidentiality. Moreover, this approach allows for the augmentation of the process data to generate new process sequences. The synthetic data could be used not only for process data analysis in various application fields, but also for enhancing the PM research, such as the development of workflow model discovery algorithms [39].

**Fig. 1.**
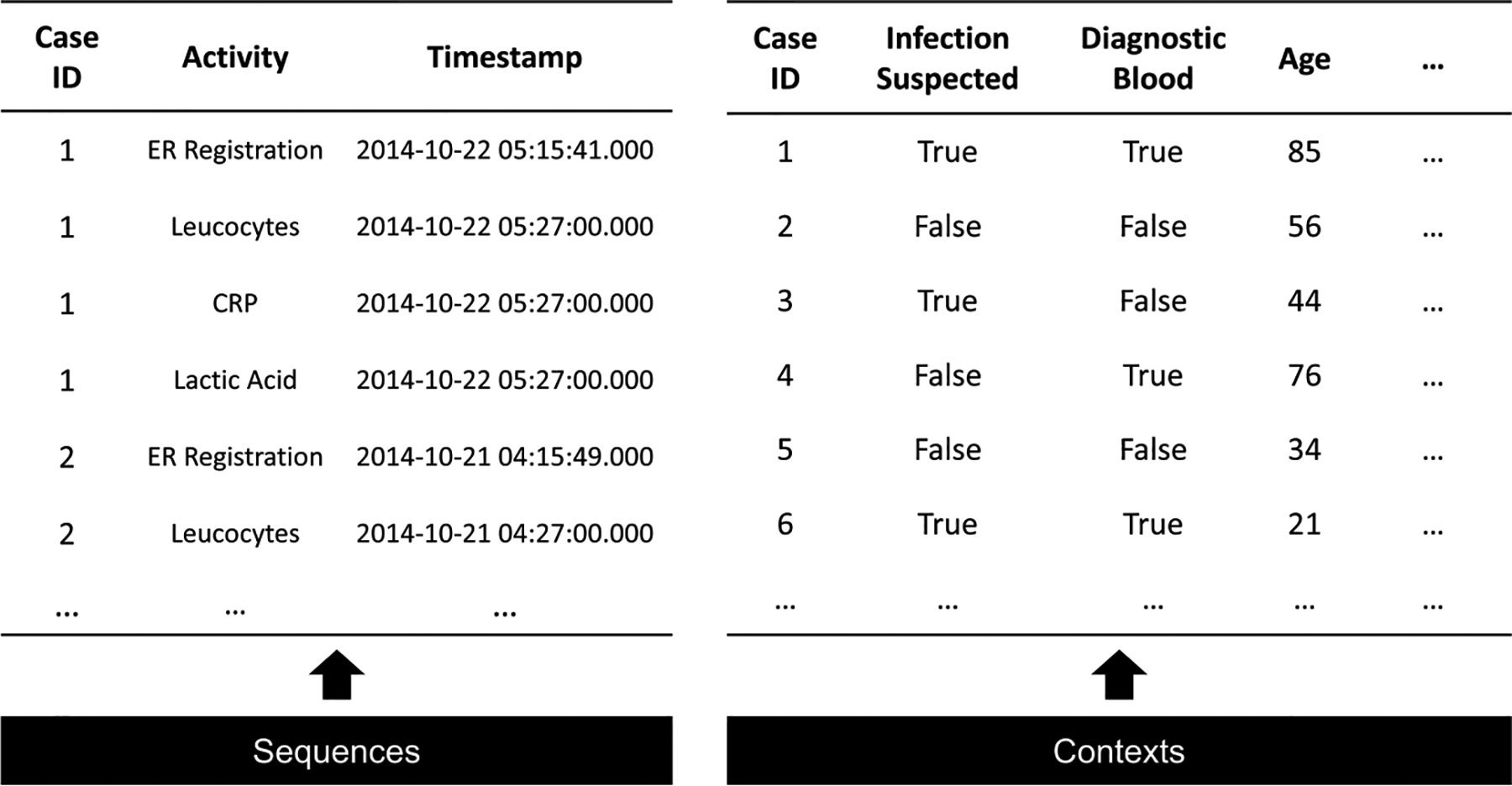
Example of an event log of the sepsis process dataset [24]. Each case in the dataset is indexed by a unique case id. A process case contains a list of performed activities, with corresponding occurring timestamp. Each case also has associated contextual features.

Process data are a sequential data constructed from a series of activities, timestamps and contexts. It is usually stored in the form of event log (Figure 1). Unlike traditional sequential data like text, process data has more attributes such as timestamps and contexts. For example, the corresponding timestamp sequence for an activity trace $x_{a}=\left[a_{1},a_{2},\ldots a_{l}\right]$ is $x_{ts}=\left[ts_{a_{1}},ts_{a_{2}},\ldots ts_{a_{l}}\right]$, where $ts_{a_{i}}$ represent the start or complete time of activity $a_{i}$. Timestamps play an important role in process analysis. For example, in trauma resuscitation cases, different time intervals between two activities might result in different subsequent treatments and patient outcomes. The time interval between patient arrival to transfusion might depend on the blood product delivery from the blood bank. A delay in the transfusion might result in higher morbidity or mortality. Process data varies according to use cases or contexts. Simple processes can be almost linear (i.e., single path) with only a few and non-repeated activities, while complex processes can have parallel paths, loops, intermediate dependencies, and hundreds of different activities. Timestamps for activities also vary according to the length (i.e., duration) of the process and may be related to the occurrence of all other activities in the sequence. For example, the same type of activity or activity pattern may occur at very different timestamps in different cases. The start time of an activity or activity pattern is based on the context of the case and is not necessarily proportional to the case duration. The occurrence of one activity may also impact the timestamps of other activities in the process. To generate timestamps for each activity, the process generation model should consider the occurrence and timestamps of all other activities and the case duration.

Recent years have seen a surge in the exploration of deep generative models for sequential data. **Generative adversarial network (GAN)** models, including SeqGAN [47] and LeakGAN [13], have proven effective in natural language generation, while transformer-based models such as GPT [30] and BERT [7] have also achieved great success. In the realm of numerical data, models like TTS-GAN [22], TimeGAN [46], and MTGAN [23] have been successfully used for time-series data generation. Although these models account for the temporal aspects of each token in the sequence, they are tailored for generating sequential data of a single type, either numerical or categorical, without producing precise timestamps for each token. We bridged this gap by generating both categorical activity sequences and numerical timestamp sequences. Our work establishes benchmarks for the synthetic process data generation task.

We aimed to generate synthetic process data that address different degrees of complexity. We focused on (1) generating plausible synthetic process data with few process errors, (2) learning the authentic data distribution when data size is small, (3) generating synthetic process data with random noise as inputs to preserve the privacy of authentic data, and (4) generating new process traces that are plausible based on the actual process. For timestamp generation, we considered (1) the duration of the sequence and (2) all other activities that occurred in the sequence. We propose ProcessGAN, a conditional GAN-based model [26], to generate synthetic process data. ProcessGAN consists of a transformer encoder-based generator and a time-aware discriminator. The transformer encoder-based generator uses the multi-head self-attention mechanism to address long-term dependencies, and the time-aware discriminator learns the attention between time-intervals, timestamps, and activities and guides the generator to generate better timestamp sequences. Unlike the existing generative models that only used temporal information between the activity tokens, ProcessGAN is a conditional GAN model that takes a predefined total duration as conditional input and outputs the activity and the timestamp sequence that reflect this inputted duration. For example, given a duration of 20 minutes, the ProcessGAN model will generate both the activity and timestamps that correspond to this time frame. ProcessGAN also retains the advantage of conventional GAN model, which can generate variant and unobserved processes from random noise.

**Fig. 2.**
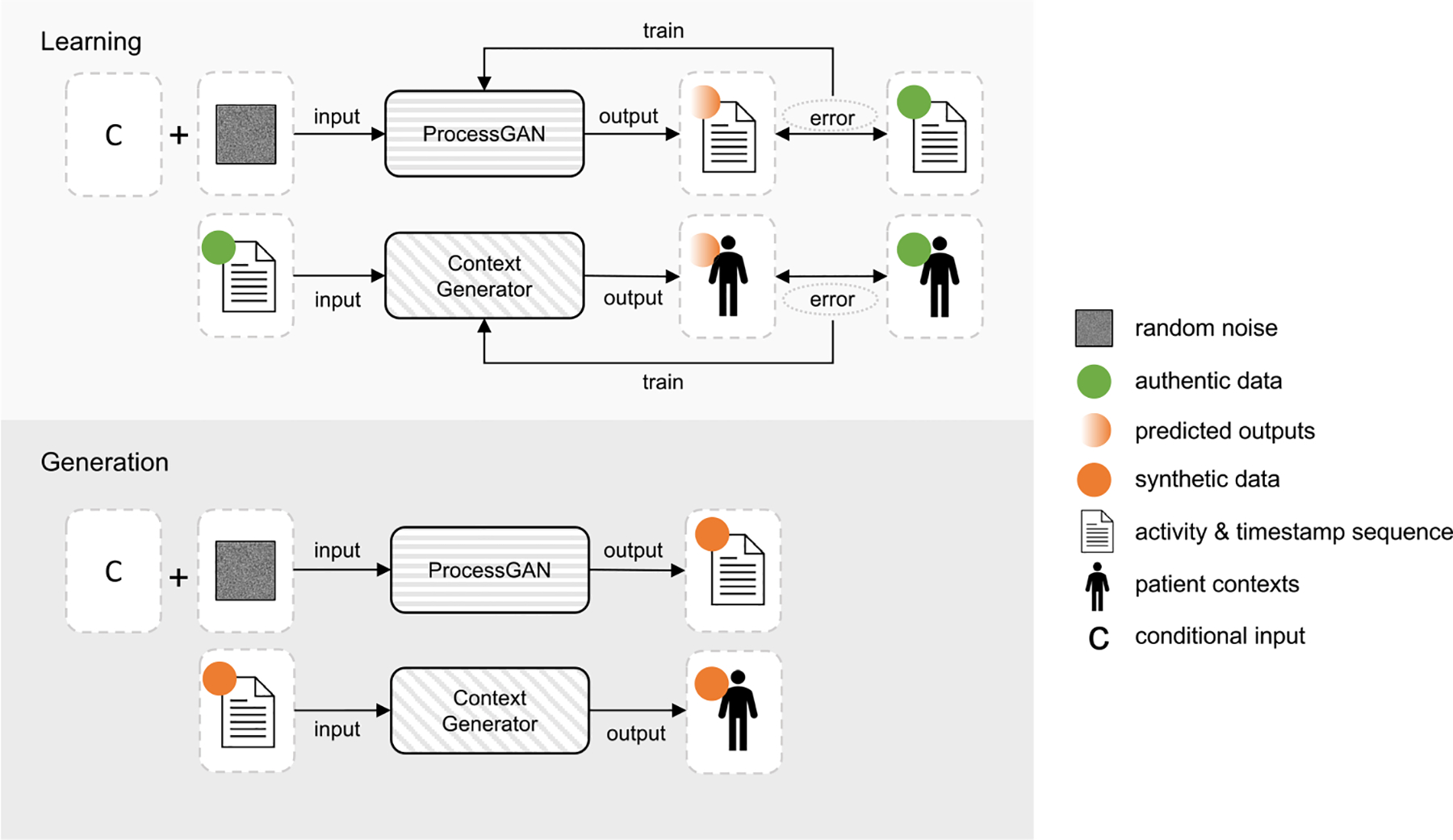
Our framework of learning and generating process data. During the learning phase, we employ adversarial training to train ProcessGAN on authentic process traces and employ supervised learning to train a context generator, which learns the correlation between process traces and contextual features. In the generation phase, the ProcessGAN model produces synthetic process traces (comprising activity and timestamp sequences) based on random noise and conditional inputs. Subsequently, the context generator utilizes these synthetic process traces to predict the most probable contextual features.

In addition to activity and timestamp sequences, process data also contains underlying contexts for each process trace (Figure 1). For example, patients with different demographics and physical conditions might need different treatment processes. The contexts associated with the process traces are an essential part of a synthetic process dataset. We propose a transformer-based context generator which learns the association between the authentic processes and contexts and generates synthetic contexts for the process traces generated by ProcessGAN (Figure 2).

We evaluated the synthetic data through three aspects: (1) statistical measurements which measure the difference between the distributions of synthetic and authentic data, (2) supervised learning score which trained a data-driven off-the-shelf model to score the synthetic data, and (3) PM which visualized the process data as workflow diagrams and allowed the knowledge-based assessment of process semantics [20]. We performed a case study on a medical process dataset and had a domain expert check if process errors occurred in the synthetic processes.

The main contributions of this article are:

- A framework for generating privacy-preserving process data which includes activity sequence and the corresponding timestamp sequence. We compared different models and analyzed their merits. Our work established benchmarks for the process data generation task.
- A novel GAN-based network called ProcessGAN for generating realistic process data. In our ProcessGAN model, the transformer-based generator takes random noise as input, case duration as conditional input, and outputs the activity and timestamp sequences. The time-aware discriminator distinguishes the authentic and synthetic process data based on the attention across activities, timestamps, and time intervals between activities.
- A context generation model based on a transformer to generate synthetic context data associated with the generated process traces. Our context generator is a multi-task model which learns to simultaneously generate different types of contexts.
- A solution for evaluating the synthetic process data. The solution includes three different dimensions: (1) statistical measures, (2) supervised learning score, and (3) PM.

## 2 Related Word

### 2.1 Time-Aware Process Data Generation

Process data can be difficult to collect and share because of underlying complexity and inclusion of private information [42]. To augment process datasets, hidden Markov models have been used to capture the sequential dependencies of activities and generate synthetic process data [45]. Studies focused on generating time-aware process data that contain both activity and timestamp sequences are still limited. Some GAN-based models have been introduced to generate synthetic sequential treatment trajectory data or predict treatment procedures [23, 34]. Although temporal information is considered in these models, each only generates either categorical event sequences or numerical time-series data. Other works have generated both categorical and numerical data such as tabular data [8, 44], but the generated data do not contain sequential information. Other research on generating event logs and timestamps [2, 6] applied **long short-term memory (LSTM)** and GAN structure to generate synthetic data for smart home devices. This model only generated these two types of data directly from the generator without considering the relations between activity type and timestamps, such as the total duration and the time-intervals.

Deep learning models such as the **recursive neural network (RNN)**-related models, transformer models, and GANs, were applied in the PM field to predict process events [3, 21, 28, 38]. Recommender systems for sequential data exist that consider both the interactions between time-interval information and the occurrence of the past items to give recommendations to the users [9, 19]. Both the absolute and relative positions between the tokens contributed to the learning of sequences [35]. These predictive models can also learn the representation of the underlying distribution of the sequential process data. The difference is that the prediction task usually applies the teacher forcing mechanism to perform the training and prediction (i.e., using the activities in the ground truth sequences to predict the next activity). In the data generation task, we do not have access to ground truth sequences. The next token is generated based on the previous generated tokens. In the case of some long complex processes, these traditional auto-regressive sequential models may suffer exposure bias [1, 31], i.e., errors occurring in the generated tokens propagate during data generation. If a sequence is generated from an unobserved starting token, divergence may occur and become enlarged along the autoregressive generation process as each token is predicted based on previously predicted tokens. These errors may reduce the authenticity of synthetic process data. In addition, the traditional **maximum likelihood estimation (MLE)** training of these networks leads to overfitting to the exposed data distribution and would not explore the vast output space beyond the exposed data distribution [31].

Unlike the traditional autoregressive models where the current token highly depends on previous tokens, the transformer network uses the positional encoding technique to inform the relative positions of tokens [41]. For this reason, the generation is more robust to the initial inputs and can generate all tokens of a sequence in parallel. This non-autoregressive step alleviates the accumulated discrepancies when tokens are generated one by one and can handle longer, more complex sequences. In our work, we developed a non-autoregressive training (i.e., generating all tokens in a sequence in parallel) of the transformer for better synthetic process data generation through GANs. With GAN-based training, feeding the transformer with random sequences as input can regularize the entire model and mitigate exposure bias.

### 2.2 GANs

Our idea of generating sharable synthetic data were inspired by the concept of image data augmentation in machine learning [36]. GAN-based neural networks have been trained on limited authentic data to enhance data size while capturing the features and generating plausible images using only random noise [10]. When using GAN, a discriminative network D was trained to distinguish between authentic and generated synthetic data, while a generative network G was trained to generate synthetic data from random input and tricks D. G gradually learned to mimic the features of the authentic data and to generate synthetic similar to actual data. This high-quality synthetic data can be used to augment training dataset for machine learning and can preserve privacy. After the vanilla GAN, conditional GAN was introduced to generate data that is conditioned by a given label [26]. Conditional GAN can direct the data generation based on the additional information.

GAN-structured generative networks have also been applied for text data generation [13, 29, 47, 48, 50]. In these works, generators are usually RNN-based models, and discriminators are usually **convolutional neural network (CNN)**-based or RNN-based models. Because the gradients from the discriminator cannot back-propagate through discrete variables, SeqGAN was introduced that uses RL [47]. Each time a new token is generated, G simulates the whole sequence and lets D score the sequence, D gives an RL reward to that token [14, 47]. With the RL reward given by D, some error tokens will have lower scores and will be less likely to be generated. G then learns the intermediate dependencies of the tokens and generates high-quality sequences. Variants of SeqGAN model such as LeakGAN address the issue of long text generation, and is the state-of-the-art text generation model for multiple benchmark datasets [13].

Although the RL rewards given by the GAN discriminator can reduce errors, the training of these GANs is difficult. The discriminator’s high variance of RL rewards may easily confuse the generator. The training process may experience mode collapse because the generator cannot learn a stable representation from the discriminator [18]. These problems are obstacles for sequential data generation, especially for complex process data that contains categorical and numerical tokens.

We addressed these challenges by replacing the RNN-based generator with the transformer-based generator which allows non-autoregressive training for better generative performance. Based on the idea of time-interval aware recommendation systems [19], we applied a multi-head self-attention network that considers the interactions between timestamps, time-intervals, and activities as the discriminator of GAN. Our model can then generate time-aware process data from random noise.

## 3 Method

In this section, we presented three key components: (1) the introduction of the ProcessGAN model, (2) the description of the context generator, and (3) the methods employed to assess the quality of synthetic process data.

**Fig. 3.**
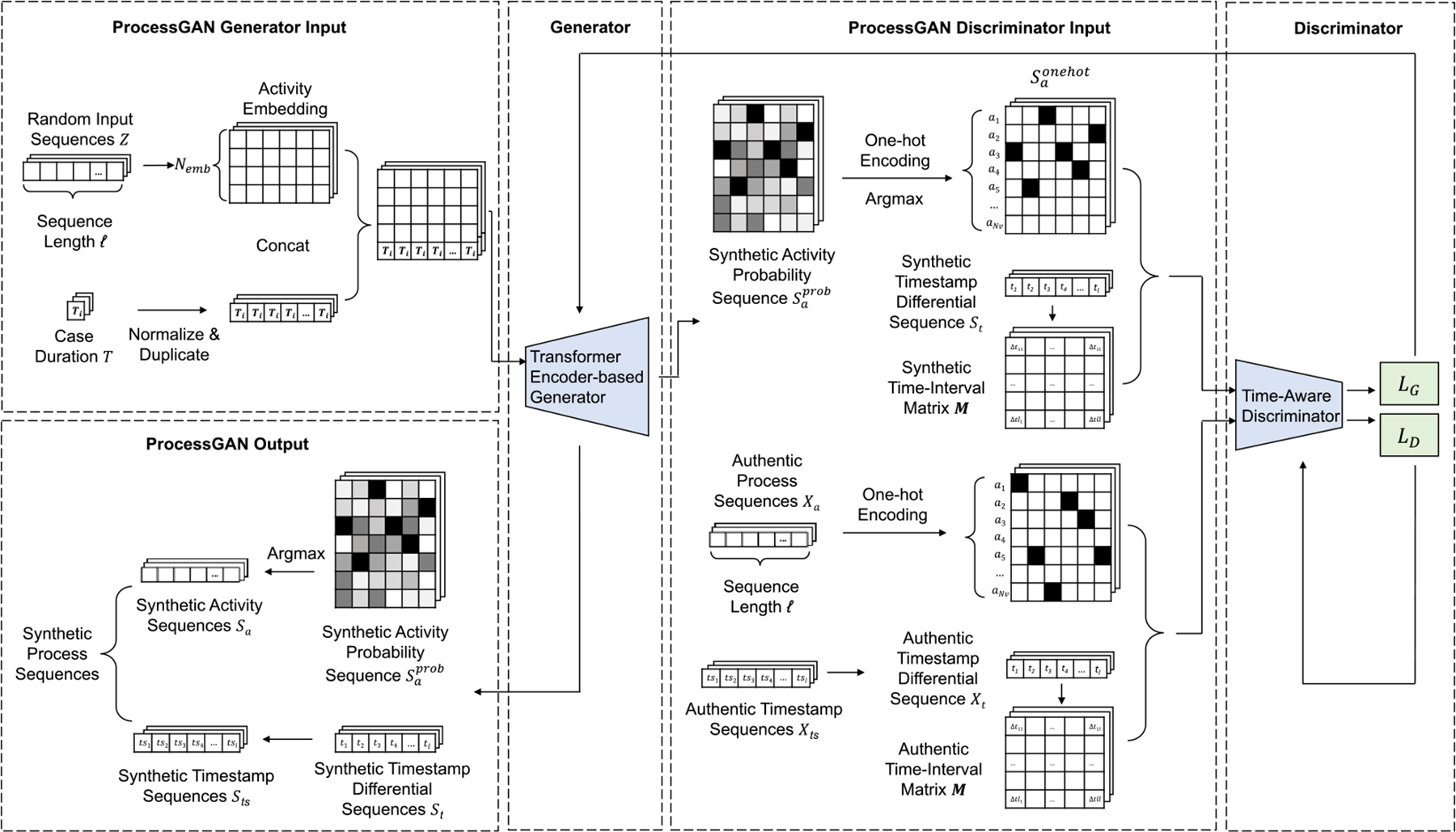
The architecture of our ProcessGAN network. The input to the generator were the random activity sequence Z, and conditional duration T that sampled from authentic processes. For each input sequence, we concatenated the input duration $T_{i}$ with the embedding of each activity. The generator’s outputs were synthetic activity probability sequence $S_{a}^{\text{prob}}$ and synthetic timestamp differential sequence $S_{t}$. For the discriminator’s input, the synthetic activity probability sequences were processed into one-hot representation. The synthetic timestamp differential sequences were processed into time-interval matrix. The activity, timestamp and time-interval features were used to train the time-aware discriminator. For the final output, the synthetic activity probability sequences $S_{a}^{\text{prob}}$ were processed into categorical activity sequence $S_{a}$ through argmax. The timestamp differential $t_{i}$ in the generated $S_{t}$ were mapped into the exact timestamp ${\text{ts}}_{i}$ for each activity.

Our ProcessGAN (Figure 3) used the transformer encoder as the generator G and a time-aware multi-head self-attention model as the discriminator D. The input to G comprised a random sequence and a conditional process duration sampled from authentic data. G produced two distinct outputs: (1) an activity sequence represented by a sequence of categorical tokens, where each token signifies the most likely activity occurs at that position, and (2) a timestamp sequence represented by a numerical sequence, where each token represents the timestamp corresponding to the respective activity position. Subsequently, the synthetic and authentic timestamp sequences underwent transformation into time-interval matrix, which encapsulated the pairwise time intervals between all activities. This matrix, along with the timestamps and activity sequences, was fed into the discriminator. During the training phase, D learned to discriminate between authentic and synthetic inputs with auxiliary objectives to reduce the divergence in activity and timestamp distributions. The auxiliary objectives served as guides for adversarial learning.

For the context generator, we employed a transformer encoder to learn the process-related features. This model was equipped with multiple “heads,” each focusing on learning the relationship between the sequence and one of the contexts.

Lastly, we proposed several approaches for evaluating the synthetic process data quality from diverse perspectives. These evaluation methods include data statistics, supervised learning, and PM techniques.

### 3.1 ProcessGAN Model

#### 3.1.1 Generator.

##### Generator Input.

The transformer-based generator G took random sequences Z as input and process durations T as conditional input. Random input sequence $z=\left[a_{1},a_{2},\ldots a_{\ell }\right](z\in Z)$ was generated by random sampling of the activity vocabulary V until the max sequence length ℓ (set to the max sequence length of the authentic data) was reached. The activity vocabulary V, with size $N_{v}$, was built from the activities $a_{i}$ in the authentic process dataset. The conditional input, process durations T, were sampled from the authentic process durations $T_{i}$. The authentic process duration $T_{i}$ was normalized into range [0, 1] based on the shortest and longest duration in the dataset ($T_{i}=\frac{T_{i_{\text{r}}\text{eal}}-T_{\text{min}}}{T_{\text{max}}-T_{\text{min}}}$).

##### Generator Architecture.

**Fig. 4.**
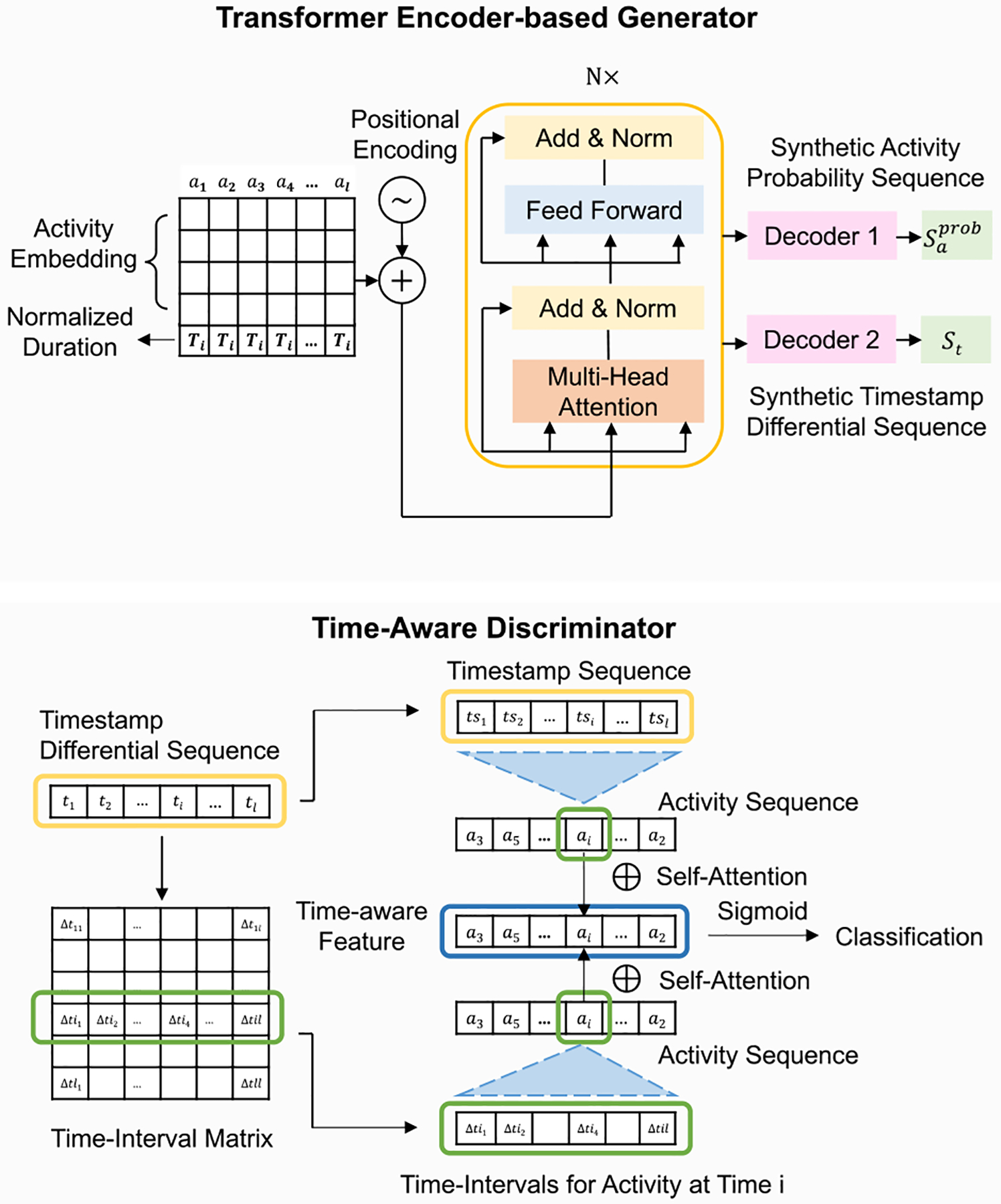
The architectures of our transformer encoder-based generator and time-aware discriminator. The generator has N self-attention blocks, and two separate decoders for generating the synthetic activity probability sequence and timestamp differential sequence respectively. Through adversarial training, it learns the dependencies between activity and timestamps, and generates synthetic data from random activity sequence and case duration (i.e., the conditional input). The discriminator uses the time-aware self-attention layers to learn the weights of activity, timestamp, and time-interval. The weights are used to generate a time-aware feature F. Given F, the discriminator then classified the input activity and timestamp sequences as positive or negative.

The generator G (Figure 4) consisted of an activity embedding layer, a transformer encoder, and two **multi-layer perceptron (MLP)** decoders. For the random activity sequences Z, we embedded the activities with an empirical embedding size $N_{emb}=\sqrt[{2}]{N_{v}}$. The transformer encoder used the self-attention mechanism to learn each token’s position and correlations with other tokens. The rich dependencies between the activities and duration in a process were captured. After the transformer encoder, we linked two separate decoders for generating the synthetic activity probability sequence and timestamp differential sequences respectively.

The output activity probability sequences were represented by categorical vectors $s_{a}^{\text{prob}}=\left[h_{1},h_{2},h_{3},\ldots ,h_{l}\right]$, where $h_{i}\in R^{N_{v}},s_{a}^{\text{prob}}\in R^{\ell \times N_{v}}$, where each vector represents a probability distribution over the activities in the vocabulary list. We applied argmax and one-hot encoding on $S_{a}^{\text{prob}}$ to obtain the one-hot representations of activity sequences, where: $s_{a}^{\text{onehot}}=\left[o_{1},o_{2},o_{3},\ldots ,o_{l}\right],o_{i}=\text{onehot}\left(\text{arg}\;\text{max}\;h_{i}\right),o\in R^{N_{v}},s_{a}^{\text{onehot}}\in R^{\ell \times N_{v}}$. The output timestamp differential sequences were represented by numerical values $s_{t}=\left[t_{1},t_{2},t_{3},\ldots ,t_{t}\right]$, where $t_{i}$ represents the differentials of timestamp from the preceding activity (we will introduce the details of the timestamp differentials later in Section 3.1.2). Because the differentials should be positive, we applied a ReLU function on the sequence and then normalized the values to fall into [0, 1] (i.e., $t_{i}=\frac{t_{i}-t_{\text{min}}}{t_{\text{max}}-t_{\text{min}}}$). In the training phase, both one-hot synthetic sequence and synthetic timestamp differential sequences were fed into the discriminator for learning. In the generation phase, we converted $S_{a}^{\text{prob}}$ to activity symbol to get the generated synthetic activity sequence $S_{a}$. For timestamp sequences $S_{ts}$, we first de-normalized the conditional input $T_{i}$ back to a real duration (i.e., $T_{i\_\text{real}}=T_{i}\times \left(T_{\text{max}}-T_{\text{min}}\right)+T_{\text{min}}$), and then mapped each timestamp differential $t_{i}$ into the exact timestamp ${\text{ts}}_{i}$ for each activity (i.e., ${\text{ts}}_{i}=T_{i\_\text{real}}\times \sum_{r=1}^{i}t_{r}$).

#### 3.1.2 Discriminator.

##### Discriminator Input.

The synthetic and authentic process data were fed into the discriminator. The process data were represented by three types of features: (1) activity sequence, (2) timestamp sequence, and (3) time-interval matrix.

Synthetic activity sequence came from the generator in one-hot format. The authentic activity sequence $x_{a}=\left[a_{1},a_{2},\ldots a_{n}\right]$ was first padded to the maximal sequence length ℓ with a predefined token [pad], and then converted to one-hot format.

The synthetic timestamp differential sequence was output from the generator and ready to be used. The authentic timestamp sequence $x_{ts}=\left[{\text{ts}}_{a_{1}},{\text{ts}}_{a_{2}},\ldots ,{\text{ts}}_{a_{n}}\right]$ was processed into timestamp differentials $x_{t}=\left[t_{a_{1}},t_{a_{2}},\ldots ,t_{a_{n}}\right]$. Given that the timestamps should be monotonically increasing, we used the positive differentials from the timestamp of the preceding activity to represent the timestamp differential sequence $x_{t}$. For each token $t_{a_{i}}$ in $x_{t}$, we normalized it into the scale of [0, 1] based on the duration of the process (i.e., $t_{a_{i}}=\frac{{\text{ts}}_{a_{i}}-{\text{ts}}_{a_{i-1}}}{T_{i_{-}\text{real}}}$, and $t_{a_{1}}=0$). Similar to the activity sequence, we pad $x_{t}$ with zeros until the length reached ℓ.

Time-intervals served as a crucial input feature for our time-aware discriminator. In process data analysis, varying time intervals between two activities could yield different sequences of subsequent activities. Our discriminator, by differentiating processes based on time-intervals, can better guide the generation of both activity and timestamp sequences. We defined time-intervals as the pairwise differentials between one activity to all other activities in the sequence. For each process case, we used a time-interval matrix $\text{M}\in N^{\ell \times \ell }$ to represent the intervals between all activities in the sequence. Given the authentic timestamp differential sequence $x_{t}=\left[t_{a_{1}},t_{a_{2}},\ldots ,t_{a_{\ell }}\right]$ where each token was the scaled timestamp differential from the previous timestamp, we first restored the scaled timestamp sequence $x_{ts}^{\prime }=\left[{\text{ts}}_{a_{1}}^{\prime },{\text{ts}}_{a_{2}}^{\prime },\ldots ,{\text{ts}}_{a_{f}}^{\prime }\right]$ by calculating the cumulative summation for each position (i.e., ${\text{ts}}_{a_{i}}^{\prime }=\sum_{r=1}^{i}t_{a_{r}}$). The time interval $\Delta t_{ij}$ between two activities $a_{i}$ and $a_{j}$ was $\left|{\text{ts}}_{a_{i}}^{\prime }-{\text{ts}}_{a_{j}}^{\prime }\right|$. By calculating the pairwise time interval between all activities in the sequence, we obtained a time interval matrix $M\in N^{\ell \times \ell }$, where

$$M=\left[\begin{array}{ccc} \Delta t_{11} & \cdots & \Delta t_{1\ell }\\ \vdots & \ddots & \vdots \\ \Delta t_{\ell 1} & \cdots & \Delta t_{\ell \ell } \end{array}\right].$$

##### Discriminator Architecture.

We modeled the time-aware discriminator D based on the interactions among input features, i.e., activity, timestamp, and time-intervals (Figure 4). Given an input activity sequence $x_{a}=\left[a_{1},a_{2},\ldots ,a_{\ell }\right],x_{a}\in R^{\ell \times N_{v}}$, we computed a time-aware feature $F=\left[f_{1},f_{2},\ldots ,f_{\ell }\right]$ for D to classify the given sequence as authentic or synthetic. $f_{i}$ represented $a_{i}$’s compatibility at the ith position given other features (i.e., activity, timestamp and time-intervals in other positions). The interactions were computed using the self-attention mechanism. We first used linear transformation on the timestamp sequence and time-interval matrix to get the key and value matrices for the self-attention mechanism [35]

$$x_{ts}^{k}=\left[\begin{array}{c} {\text{ts}}_{1}^{k}\\ \vdots \\ {\text{ts}}_{\ell }^{k} \end{array}\right],\;x_{ts}^{v}=\left[\begin{array}{c} {\text{ts}}_{1}^{v}\\ \vdots \\ {\text{ts}}_{\ell }^{v} \end{array}\right]$$

$$\begin{array}{c} M^{k}=\left[\begin{array}{ccc} \Delta t_{11}^{k} & \cdots & \Delta t_{1\ell }^{k}\\ \vdots & \ddots & \vdots \\ \Delta t_{\ell 1}^{k} & \cdots & \Delta t_{\ell \ell }^{k} \end{array}\right],\;M^{v}=\left[\begin{array}{ccc} \Delta t_{11}^{v} & \cdots & \Delta t_{1\ell }^{v}\\ \vdots & \ddots & \vdots \\ \Delta t_{\ell 1}^{v} & \cdots & \Delta t_{\ell \ell }^{v} \end{array}\right] \end{array}$$

$f_{i}$ in the time-aware feature sequence F is defined as the weighted sum of the linearly transformed representations of the input features

$$f_{i}=\sum_{j=1}^{\ell }\alpha_{ij}\left(a_{j}W^{V}+\Delta t_{ij}^{v}+{\text{ts}}_{j}^{v}\right),$$

where the weight coefficient $\alpha_{ij}$ is computed by softmax

$$\alpha_{ij}=\frac{\text{exp}\;e_{ij}}{\sum_{k=1}^{\ell }\text{exp}\;e_{ik}}.$$

The $e_{ij}$ computes the compatibility between the ith and jth position in the sequence based on scaled dot product:

$$e_{ij}=\frac{{\left(a_{i}W^{Q}\left(a_{j}W^{K}+\Delta t_{ij}^{k}+{\text{ts}}_{j}^{k}\right)\right)}^{⊤}}{\sqrt{d}}.$$

For activity’s query, key, and value, $W^{Q},W^{K},W^{V}\in R^{N_{v}\times d}$ are the parameter matrices to be trained through backpropagation, d is the hidden dimension of the model.

After the time-aware self-attention layer, we applied a ReLU activation to add non-linearity. We adopted layer normalization, residual connection, and dropout method to reduce overfitting and unstable training as introduced in [19]

$$f_{i}=\text{FFN}\left(\text{ReLU}\left(f_{i}+\text{Dropout}\left(\text{FFN}\left(\text{LayerNorm}\left(f_{i}\right)\right)\right)\right)\right).$$

The time-aware feature $F=\left[f_{1},f_{2},\ldots ,f_{\ell }\right],F\in R^{\ell \times d}$ was computed separately for input data, e.g., either synthetic or authentic process data. After these time-aware self-attention stacks, we applied a feed-forward layer and sigmoid function to get a classification output to determine whether the input process was synthetic or authentic.

#### 3.1.3 Learning Objectives.

The generator and discriminator were optimized alternatively to achieve an equilibrium. The discriminator was used to score the generated sequences and train the generator, while the generator’s objective was to maximize this score. The activity sequences output from the generator were the probability distribution over each activity type. We extracted the most likely activities from the sequence and built a synthetic activity sequence. In the forward propagation, we applied argmax on $S_{a}^{\text{prob}}$ to generate a sequence in one-hot encoding representation, which was then fed into the discriminator. The argmax operation cannot be differentiated in the back propagation, and the gradient cannot be passed back. We hence adopted the straight-through Gumbel-softmax mechanism with a differentiable sampling operation [14]. To achieve differentiable approximation, Gumbel-softmax (1) used Gumbel-max trick to refactor the sampling function by introducing independent noise from a Gumbel distribution; and (2) used softmax as a differentiable approximation to argmax. The discriminator scored the generated sequences and passed the gradient back to the generator. The loss function of the conditional generator was

$${ℒ}_{G}=-E_{Z\sim P\left(Z\right)}\;\text{log}\;D\left(G\left(Z|T\right)\right),$$

where: $G(Z|T)=S_{a}^{\text{prob}},S_{t}$, and $T\in T_{i}$.

The discriminator distinguished between the generated and the authentic processes. The loss function for the discriminator was

$${ℒ}_{D}=-E_{X\sim P_{\text{data}}}\;\text{log}\;D\left(X|T\right)-E_{Z\sim P\left(Z\right)}\;\text{log}\;\left(1-D\left(G\left(Z|T\right)\right)\right).$$

Compared with RNNs, the random inputs and adversarial training in ProcessGAN may reduce exposure bias. Due to the larger search space, it is harder for the ProcessGAN to converge [12]. To help reduce the search space, we added activity and timestamp distribution divergences as two auxiliary losses to the generator. The activity distribution divergence was defined using MSE, where $X\prime_{a}\left(a_{i}\right)$ and $S\prime_{a}\left(a_{i}\right)$ are the activity frequency of $a_{i}$ in the authentic and synthetic dataset, respectively. The loss was defined as

$${ℒ}_{G}^{a}=\text{MSE}\left(X\prime_{a},S\prime_{a}\right)=\frac{1}{m\times N_{v}}\sum_{i=1}^{N_{v}}{\left[X\prime_{a}\left(a_{i}\right)-S\prime_{a}\left(a_{i}\right)\right]}^{2},$$

where m is the batch size, $X\prime_{a}$ and $S\prime_{a}$ are the activity probability distributions of one batch of authentic sequences and generated sequences. The divergence in timestamp distribution was computed using the MSE of the mean values of each activity’s timestamps in the dataset, where $X\prime_{ts}\left(a_{i}\right)$ and $S\prime_{ts}\left(a_{i}\right)$ represent the mean timestamps of activity $a_{i}$ in the authentic and synthetic dataset, and

$${ℒ}_{G}^{t}=\text{MSE}\left(X\prime_{ts},S\prime_{ts}\right)=\frac{1}{m\times N_{v}}\sum_{i=1}^{N_{v}}{\left[X\prime_{ts}\left(a_{i}\right)-S\prime_{ts}\left(a_{i}\right)\right]}^{2}.$$

**Algorithm 1:** Compute Expectations of Adversarial and Auxiliary Loss

1:	**Input:** Random activity sequence Z, process duration T, authentic activity and timestamp sequence $X_{a}$, *$X_{t}$*, and the synthetic activity and timestamp sequence $S_{a}$, *$S_{t}$*. Number of epochs e.
2:	**Output: $E\left({ℒ}_{G}\right),E\left({ℒ}_{G}^{a}\right),E\left({ℒ}_{G}^{t}\right)$**
3:	**for** _ in range(e) **do**
4:	$S_{a}$, *$S_{t}$* ← g_model(Z,T)
5:	$E\left({ℒ}_{G}\right)$ **+=** cross_entropy(d_model($S_{a}$, *$S_{t}$*), label **=** 0)
6:	$E\left({ℒ}_{G}\right)$ **+=** cross_entropy(d_model($X_{a}$, *$X_{t}$*), label **=** 1)
7:	$E\left({ℒ}_{G}^{a}\right)$ **+=** mse(syn_act_freq, aut_act_freq)
8:	$E\left({ℒ}_{G}^{t}\right)$ **+=** mse(syn_time_mean, aut_time_mean)
9:	**end for**
10:	$E\left({ℒ}_{G}\right)\leftarrow E\left({ℒ}_{G}\right)/e$
11:	$E\left({ℒ}_{G}^{a}\right)\leftarrow E\left({ℒ}_{G}^{a}\right)/e$
12:	$E\left({ℒ}_{G}^{t}\right)\leftarrow E\left({ℒ}_{G}^{t}\right)/e$
13:	**return $E\left({ℒ}_{G}\right),E\left({ℒ}_{G}^{a}\right),E\left({ℒ}_{G}^{t}\right)$**

We calculated the divergence in each training batch and fed it to the generator. With divergence loss (${ℒ}_{G}^{a}$ and ${ℒ}_{G}^{t}$), the generator loss became

$${ℒ}_{G}^{\prime }={ℒ}_{G}+w_{a}{ℒ}_{G}^{a}+w_{t}{ℒ}_{G}^{t},$$

where $w_{a}$ and $w_{t}$ were the weights of the auxiliary losses. $w_{a}$ and $w_{t}$ were hyperparameters that balanced adversarial loss and divergence loss. We chose the weight heuristically as a value that enforced the three losses at a similar scale. We first ran the generator several times without optimization and obtained the expectations of adversarial and divergence losses: $E\left({ℒ}_{G}\right),E\left({ℒ}_{G}^{a}\right),E\left({ℒ}_{G}^{t}\right)$ (Algorithm 1). $w_{a}$ and $w_{t}$ were then approximated as $w_{a}=\frac{E\left({ℒ}_{G}\right)}{E\left({ℒ}_{G}^{a}\right)}$ and $w_{t}=\frac{E\left({ℒ}_{G}\right)}{E\left({ℒ}_{G}^{t}\right)}$.

#### 3.1.4 Adversarial Training.

At the early adversarial training stage, D may quickly converge because G is generating implausible sequences. D may then reject the generated sequences and lead to poor optimization of G. To control the optimization speed and slow down D’s convergence, we adopted the training scheme of optimizing k epochs (k set to 2 in our experiments) of G and used one epoch of D [10]. To use this training scheme in ProcessGAN implementation, we optimized the discriminator at the nth epochs when n is divisible by k.

Determining the optimal stopping point for GAN models, especially with non-visual data like sequences, can be challenging due to the oscillating loss values in the minimax training game. The accuracy of D hovering around 0.5 signifies that G is generating plausible sequences that trick D. In our experiments, we trained the ProcessGAN model for a set number of epochs, recorded the results every 50 epochs, and selected the outcome when D and G reached the equilibrium [10]. For optimization, we used Adam optimizer for both G and D. The detailed training steps were shown in Algorithm 2.

### 3.2 Contexts Generator

**Fig. 5.**
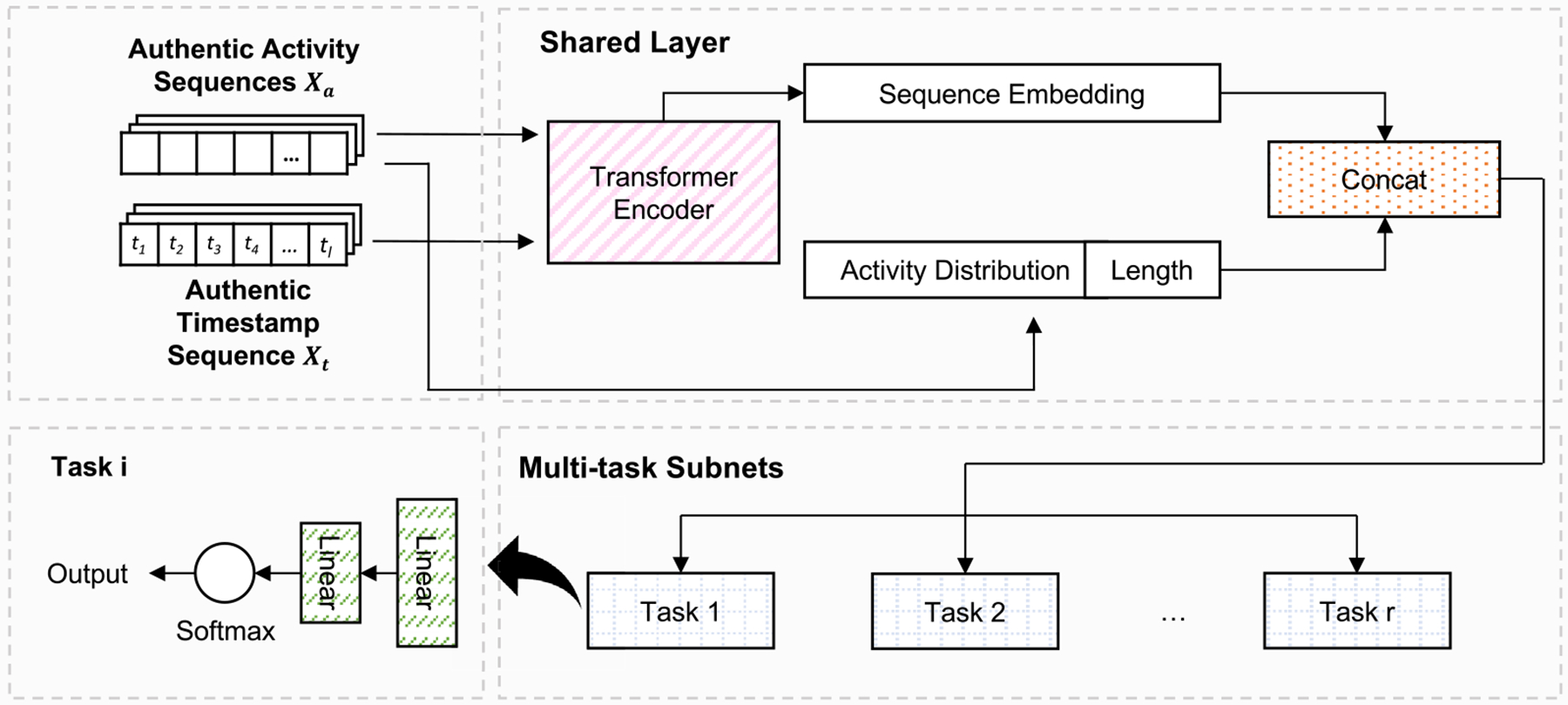
The learning process of the context generator. The process embeddings were first obtained from the shared transformer layer, then concatenated with the activity distribution and the sequence length. The features were then fed into multiple task-specific subnets to generate different contexts.

Context generator aims to first learn the associations between authentic process traces (activity and timestamp sequences) and the corresponding contextual features. It then infers the contextual features on the generated synthetic traces. The problem is formulated as a multi-task learning that predicts r different context features ($C=\left\{c_{1},c_{2},\ldots ,c_{r}\right\}$) based on the observed process traces (Figure 5).


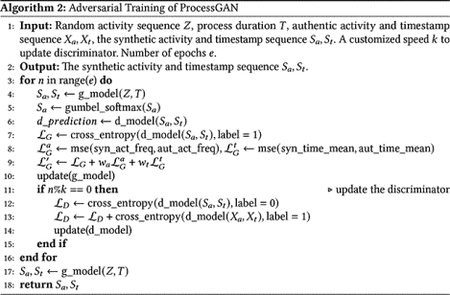


We first fed the process traces into a transformer encoder to generate the embeddings. The output sequence embeddings were then concatenated with the activity frequency distribution $\left(p_{i}=p\left(x=a_{i}\right),\left(i=1,2,\ldots ,N_{v}\right)\right)$ and sequence length to capture more sequence information. The parameters of the transformer layer were shared in the model. The sequence features were then fed into r separate output “heads” (task-specific subnets). Each output head contains two dense layers and handles one context learning task.

The contexts can be categorical or numerical. The loss value $L_{i}$ was calculated separately for each context. For categorical contexts, the subnets were classification networks. We used the cross-entropy loss ($L_{i}=L_{\text{CE}}$) to update the model. For numerical contexts, the subnets were regression networks. The mean square error loss was applied ($L_{i}=L_{\text{MSE}}$). The weighting between each task’s loss can strongly impact model performance. Tuning these weights by hand can be challenging and time-consuming. For this reason, we used the uncertainty-based (homoscedastic uncertainty) weighting method [16] which considers the homoscedastic uncertainty of each task to calculate the loss value

$$L\prime_{i}=\frac{1}{\sigma^{2}}L_{i}+\text{log}\;\sigma^{2}.$$

This method in multi-task learning assumes prediction errors have constant variance across tasks, where the parameter $\text{log}\;\sigma^{2}$ represents this variance. In the model, each task has a learnable $\text{log}\;\sigma^{2}$ that dictates its importance during training. A higher $\text{log}\;\sigma^{2}$ means the task has more uncertainty and is given less weight, while a lower value prioritizes that task. This allows the model to adjust and optimize performance for each task based on their uncertainties. The overall loss for the context generator $L_{c}$ was obtained by summing the individual losses

$$L_{c}=\sum_{i=1}^{r}L\prime_{i}.$$

We used the Adam optimizer to calculate the gradients. The model was evaluated on the authentic test set based on the F-measures. After obtaining adequate performance, we applied the model on synthetic data to obtain the context.

### 3.3 Evaluation Methods for the Synthetic Process

Evaluation of sequential data are not as intuitive as image data. Synthetic images are usually evaluated based on the authenticity of characters or the resolution, features that are easily observable [10, 15]. We aim to generate synthetic process data that follows the underlying distribution of the real-world process. Our evaluation took a sample of synthetic processes and evaluates their authenticity using three measures, i.e., (1) statistical measures, (2) supervised learning score, and (3) PM.

#### Statistical Measures.

We used four quantities to measure the error between synthetic and the authentic processes.

- *Sequence length*: The number of tokens in a sequence. We compared the mean and standard deviation of sampled synthetic and authentic processes.
- *Sequence variance*: The variance of sequences measures the spread or distance between activity sequences in a dataset. We used the ***S****um of* ***P****airwise normalized* ***E****dit distance* (SPE) to measure variance:
$$\text{SPE}=\frac{1}{N^{2}}\sum_{i=1}^{N}\sum_{j=i+1}^{N}\frac{\text{ED}\left(s_{i},s_{j}\right)}{\text{length}\left(s_{i}\right)+\text{length}\left(s_{j}\right)},$$
  where N is the number of sequences in the generated samples, s are the sequences and ED is the edit distance (Levenshtein distance).
- *Activity type occurrence error*: The error between the count of occurrence of each activity type in synthetic versus authentic data. Because each process data had different number of activity types, we reported an aggregated metric to measure the error of activity occurrence
$$\text{ActError}\left(X_{a},S_{a}\right)=\sum_{i=1}^{N_{v}}\left|X_{a}\left(a_{i}\right)-S_{a}\left(a_{i}\right)\right|,$$
  where $X_{a}\left(a_{i}\right),S_{a}\left(a_{i}\right)$ represented the activity frequency of $a_{i}$ in authentic and synthetic data, respectively. The metric measures the L1 distance between the percentages of each activity type.
- *Activity timestamps error*: The sum of each activity’s timestamp’s differences between authentic and synthetic dataset. In our experiments, we used the mean values and 90% percentile values to measure the timestamp expectation $E\left(ts_{a_{i}}\right)$ for each activity
$$\text{TimeError}\left(X_{t},S_{t}\right)=\sum_{i=1}^{N_{v}}\left|E{\left(ts_{a_{i}}\right)}_{\text{aut}}-E{\left(ts_{a_{i}}\right)}_{\text{syn}}\right|.$$
  These measurements described the basic dimensions of a process sequence. The activity type occurrence and activity timestamp distribution were cross measurements, i.e., computed across synthetic and authentic process sequences. The smaller error indicates higher affinity between synthetic data and authentic data.

#### Supervised Learning Score.

Domain experts could manually evaluate the generated processes. This manual assessment is labor-intensive and is vulnerable to bias. For this reason, we trained an independent binary classifier and used it as an “off-the-shelf” supervised scorer to assess the realism of the synthetic data. The classifier can be an evaluator with comprehensive domain knowledge that can distinguish very similar positive and negative sequences. We adopted the transformer model as the binary classifier. Although we used transformer in this work, traditional sequential classification models, like RNN, CNN, or tree models with crafted features as input, can also be used as the classifier.

**Fig. 6.**
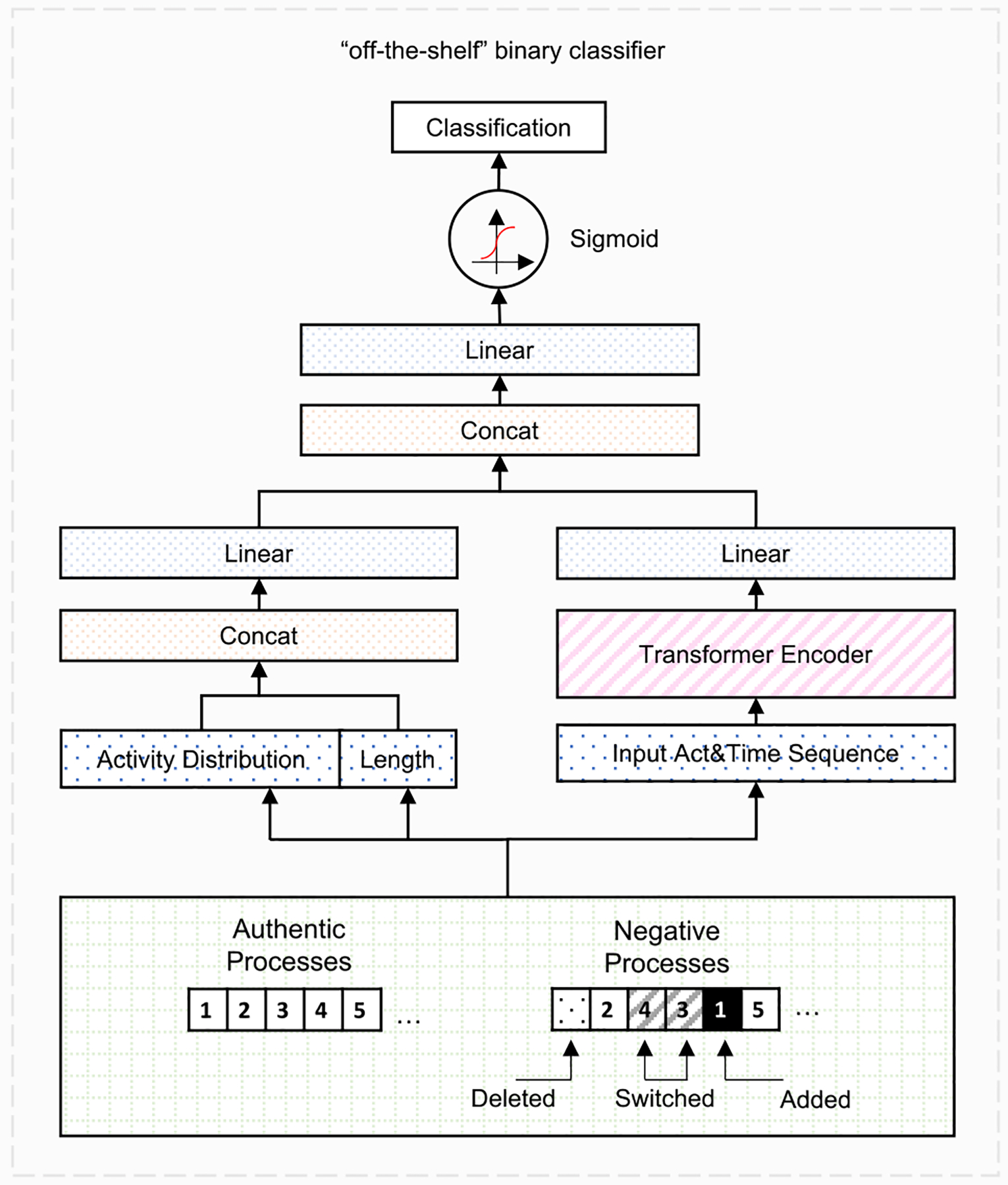
The architecture of the “off-the-shelf” evaluation model. To generate negative sequences, we randomly added noise to the authentic sequence. The input of the classifier was trained based on the activity sequences, timestamp sequences, activity distribution and sequence lengths.

Similar to the negative sampling method in the nature language processing field, we created high-quality processes to get the negative samples by manually adding noise (randomly adding, deleting, and switching a predefined ratio of tokens) to the authentic sequences [25]. For timestamps, when adding an activity, we randomly picked a timestamp between the added positions. For deleting, we deleted the timestamp with the activity. For switching, we only switched the activities and kept the timestamp the same. The timestamps were then processed into the range [0, 1]. To distinguish more data features, we concatenated each process with its activity frequency distribution and sequence length after the transformer layer and sent them to two additional dense layers (Figure 6). Let θ be the parameters of the model, the objective was to maximize the log-likelihood with respect to θ

$$\theta \to \sum_{g\in T}\text{log}\;p(y|g,\theta ),$$

where T is the training set that contains the positive and negative samples, and y is the correct class of g.

We generated negative samples five times more than the positive samples to augment the training data. After the transformer achieved a good F1 score (F1 > 0.8), we used it to score the synthetic processes. The classification score was defined as the false positive rate (FPR = FP/N) of the “off-the-shelf” model, i.e., the count of synthetic processes misclassified as authentic, and N was the count of all negatives, i.e., synthetic process traces. This score measured the fraction of the synthetic processes that the “off-the-shelf” binary classifier classified as authentic. This fraction measured how frequently the synthetic data “tricked” the binary classifier.

#### PM.

We applied the workflow discovery method from the PM field to construct a workflow diagram from the generated process traces [20, 43]. We aimed to visualize the process traces and manually evaluate whether they represented the underlying distribution of authentic data.

To construct the diagram, we used the trace alignment method to generate consensus sequence from the alignment result [5]. A consensus sequence captures the major activities in the process traces and can be considered the workflow diagram’s backbone. Some activities that are not in the consensus sequence are added as side branches in addition to the backbone to represent the parallel activities. We asked a domain expert to compare the workflow diagrams and check for process errors.

## 4 Experiments and Results

### 4.1 Dataset Introduction

We performed our experiment and analysis on three medical process datasets of pediatric trauma resuscitation (the secondary survey, the process of **intubation (INT)**, and the airway assessment). The process traces were manually coded from videos of actual events. The use of these data were approved by the Institutional Review Board of Children’s National Hospital in Washington, DC. In addition, we used two public process datasets to evaluate our method on different types of processes: a real-world event log which contains the process of sepsis cases from a hospital and an event log of a loan application process (Table 1).

### 4.2 Experimental Design

#### 4.2.1 ProcessGAN Experiments.

##### Baseline Methods.

ProcessGAN is the first generative model for process data generation. No public implementation of generative models for activity and timestamp sequence generation is available. For this reason, we applied the existing SeqGAN model [47] as a baseline model for activity sequence generation. We used SeqGAN as our baseline because it was the first GAN-structured model introduced for sequential data, and later models like LeakGAN were the variants of SeqGAN for specific use cases for language generation. SeqGAN model used an LSTM model as the generator and a CNN model as a discriminator. The generator and discriminator were first pretrained. The GAN model was then trained by adding RL reward, which was given by the CNN-based discriminator to the LSTM-based generator. Considering that SeqGAN is not applicable for timestamp (continuous data) generation, we applied random sampling from the real distribution of the timestamps at each step after obtaining the generated activities from SeqGAN. We used “SeqGAN_T_” to denote our modification to the original SeqGAN model.

##### Ablation Experiments.

We did ablation experiments to test the effectiveness of each component of the ProcessGAN model. Our experiments included:

- W/O timestamp loss: We removed the MSE loss of the timestamps to train the ProcessGAN model.
- W/O activity loss: We removed the MSE loss of the activity frequency distribution to train the ProcessGAN model.
- W/O timestamp and activity losses: We removed both auxiliary losses.
- W/O time-interval attention module: We removed the time-interval attention module and used the encoder part of the transformer model as the discriminator for the ProcessGAN model. The input of timestamp sequence, time interval matrix and activity sequence were concatenated and fed into the vanilla transformer-based discriminator.
- W/O time-interval attention module and auxiliary losses: We removed the time-interval attention module and the auxiliary losses.

We configured the ProcessGAN model based on the data sizes and vocabulary sizes between different datasets (Table 2). Our model was trained on a NVIDIA 3090Ti GPU. For each model, we generated the same number of synthetic processes as in the authentic data and applied the evaluation methods we introduced in Section 3.3.

#### 4.2.2 Context Generator Experiments.

##### Baseline Methods.

The context data in our dataset was categorical. For this reason, we applied three simple classification method as our baselines:

- Random selecting: randomly pick one of the categories from a uniform distribution.
- ZeroR: choose the majority category for the classification.
- Single-task MLP-based generator: train multiple MLP-based models on the activity frequency distribution in the traces, with each model handling only one task.

Considering the small size of our dataset, we applied five-fold cross validation for training and evaluation. We used a batch size of 16 and set the initial learning rate to 1e-2. We monitored the learning rate using a step decay scheduler that reduces the learning rate every 10 epochs. We set the parameters of the Adam optimizer to be (0.5, 0.99). We applied an early stopping after 20 updates of loss increments to avoid overfitting. The final performance was averaged over the five validation sets.

### 4.3 Results

#### 4.3.1 Quantitative Results.

##### Statistics.

For the sequence lengths, we found the ProcessGAN model outperformed the baseline model, especially for datasets with longer sequences (Table 3). The discriminator of the GAN network learned the distribution of sequence length and helped avoid generating biased sequences that were either too long or too short. For longer sequences with smaller data sizes (SS, AIR), the SeqGAN_T_ model tended to generate sequences with shorter lengths or a larger standard deviation. Although the LSTM-based generator and CNN-based discriminator were pretrained, the SeqGAN_T_ model had difficulty learning longer length sequences. We found it hardly improved the performance of the pre-trained generator. In the ablation experiments of the ProcessGAN model, we found that adding the auxiliary loss values from either activity or timestamp divergence helped the model generate more realistic sequence lengths.

From the sequence variance perspective, all models generated variable sequences (i.e., the variance of each model was > 0). The auxiliary loss from the activity frequency distribution helped generate sequences with the sequence variance similar to the authentic data.

The ProcessGAN and SeqGAN_T_ models performed well for activity type occurrence. This observation was expected because the SeqGAN_T_ model was pre-trained with the MLE objective through teacher forcing, in which case the activity distribution of the training data can be easily learned. The ProcessGAN model without activity-based auxiliary loss showed a large difference in activity type occurrence compared to authentic data. With activity distribution divergence as an auxiliary loss, ProcessGAN was able to generate activity distributions closer to authentic data. For complex processes (i.e., larger activity vocabulary) with limited data size (INT, AIR), the SeqGAN_T_ model did not have an advantage in generating an adequate activity distribution. The performance of MLE-based pretraining relied on having sufficient data to learn the distribution of each activity type. The process data complexity and sample size can impact the performance of different models. ProcessGAN model provided more regularizations when sequences were complex and when data size was insufficient, making the model suitable for process data augmentation in these conditions.

For the timestamp sequences, ProcessGAN model achieved the best performance among all datasets (Table 3). We used the authentic process durations $T_{i}$ so ProcessGAN was expected to generate similar timestamps to the authentic data. We used mean and 90% percentile value to measure the error in activity timestamps. Given a process duration as the conditional input, a lower error based on the mean value indicated that the timestamps generated by ProcessGAN for each activity were more aligned with the actual occurrence time in the authentic case of identical duration. On the other hand, the 90% percentile-based error reflected the range within each activity resides. A smaller error signified that a larger fraction of timestamps generated by ProcessGAN align well with the range observed in the authentic data. From the ablation experiments, the timestamp-based loss function and the time-aware attention module helped generate better timestamps. The loss value from activity frequency distribution also helped generate precise timestamps, which indicated that the authenticity of timestamps relied on the quality of activity frequency.

**Fig. 7.**
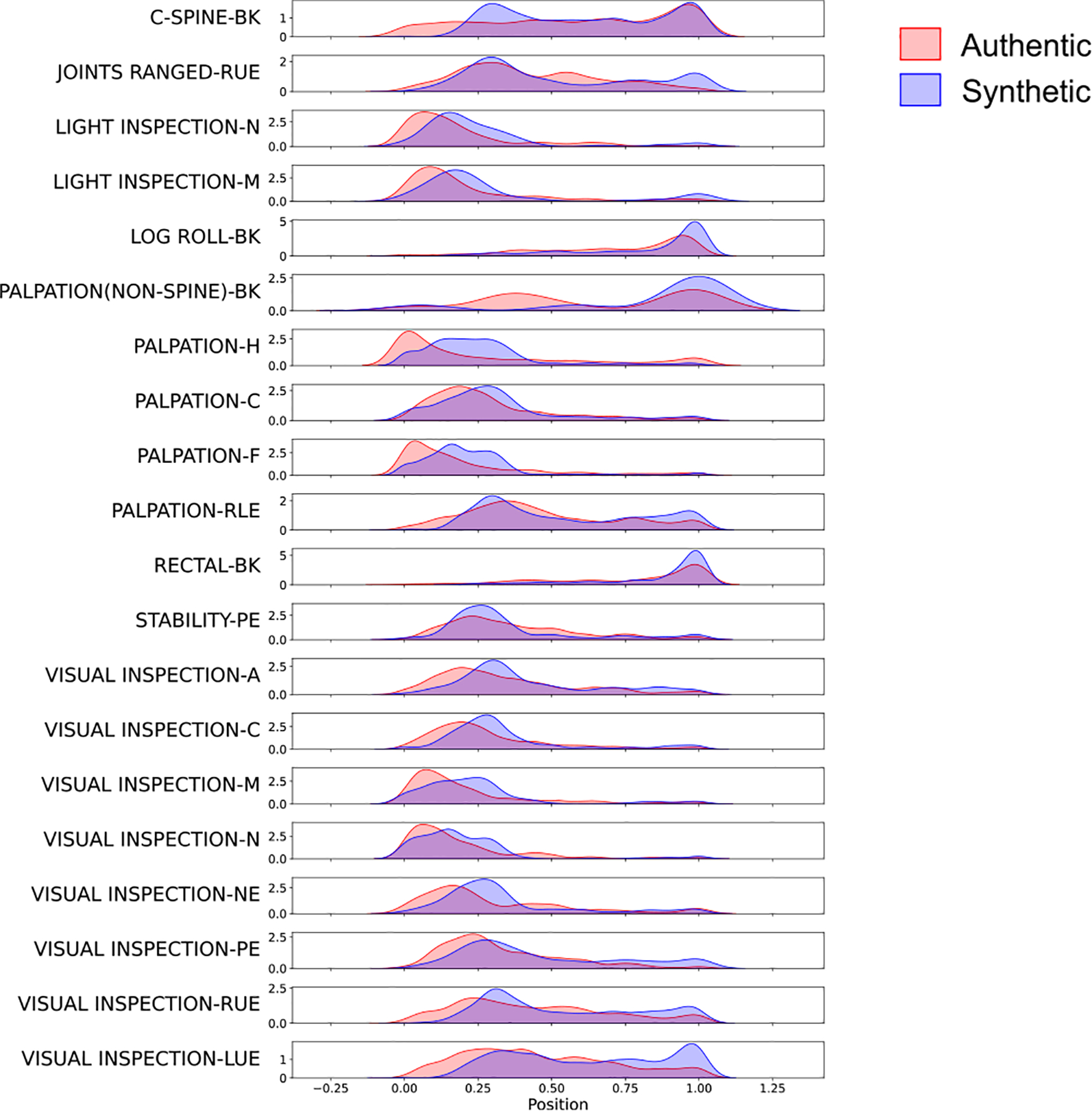
The plot of timestamp distribution for the activities that occurred in the dataset. The plot of timestamp distribution for the activities that occurred in the dataset. We plotted the results for the secondary survey dataset. The x-axis is the scaled duration of the processes in the dataset, and for each activity, the y-axis shows the density of that activity at different positions. Figure 7 compares the distributions between authentic data and ProcessGAN generated data. The results for other models are available in Appendices, Figure A1.

**Fig. A1.**
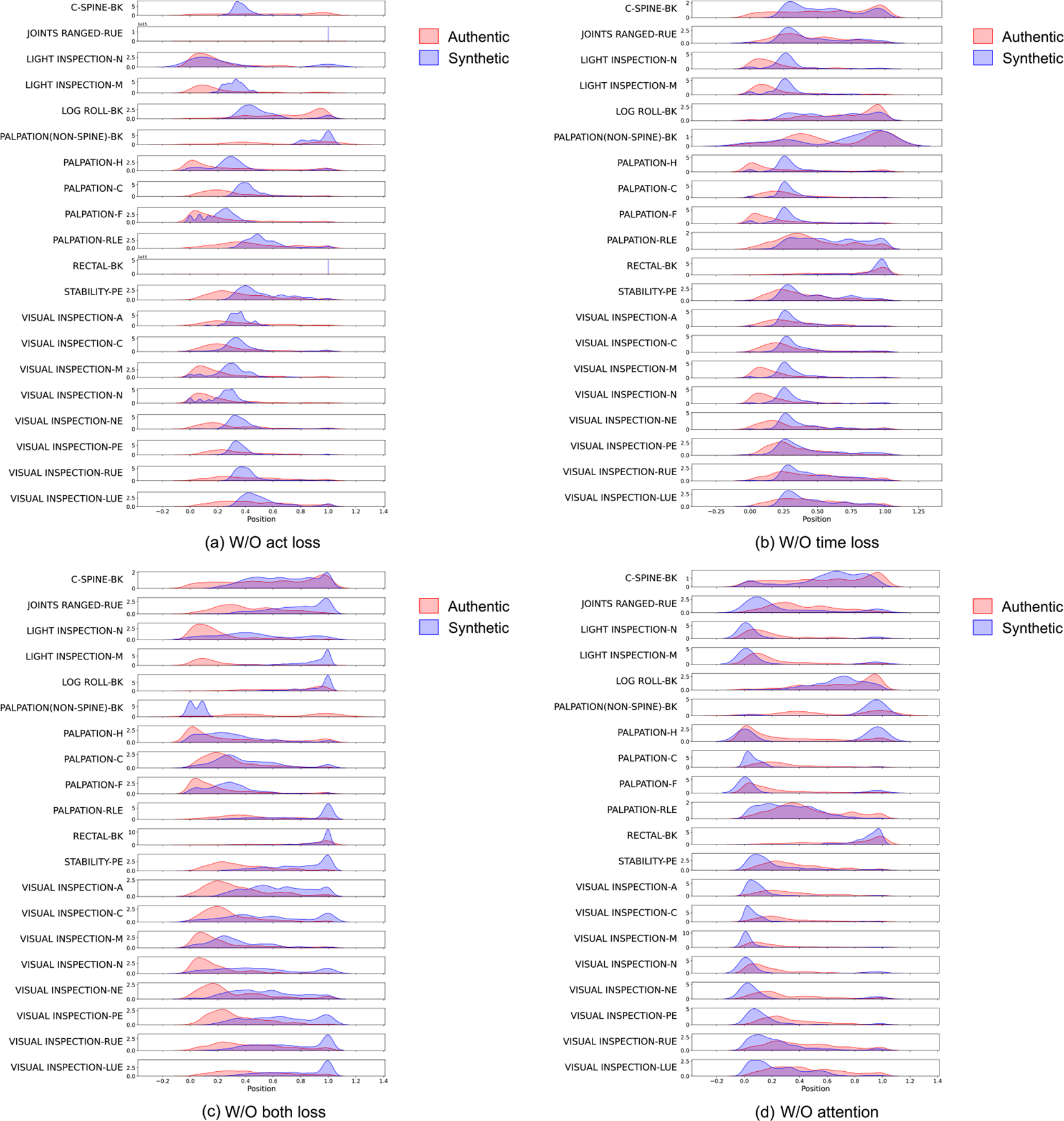

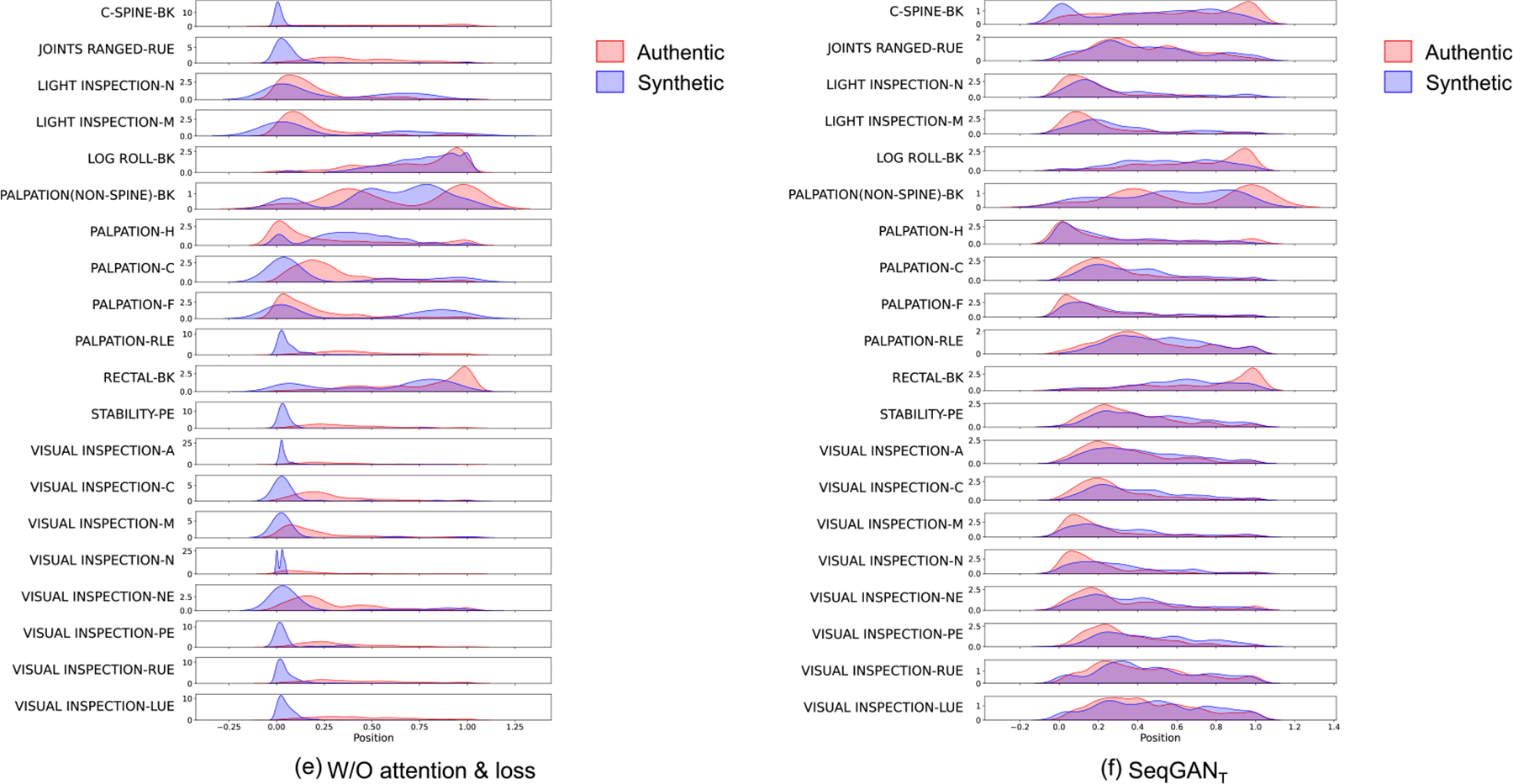
Timestamp distribution for each type of activity that occurred in the dataset. Chart A to E shows results from ablation studies. Chart F shows SeqGAN_T_ results. The blue shadows are distribution of synthetic data while the red shadows are authentic data. The x-axis is the scaled duration of the processes in the dataset, and y-axis shows the density. Note that although the visualizations of authentic data curves might be different because of the differences in y-axis’ range, the distribution of the authentic data keeps the same among all comparisons.

We used kernel density estimation curves to show the timestamp results for the secondary survey data (Figure 7). During secondary survey of the trauma resuscitation, usually the assessments of the patients’ head (-H), face (-F), and nose (-N) are performed at the beginning, followed by chest (-C), pelvis (-PE), and extremities (-LUE or -RUE), while back assessments (-BK) are usually performed last. The ProcessGAN model (Figure 7) generated the timestamp pattern that was most similar to authentic data and followed the assessment procedure. Without timestamp-based loss value (Figure A1(B)), although the maximal activity density was close to the authentic data, the timestamps of each activity were not precise. The timestamps of all the activities were more centralized without a trend that existed in the authentic data. Without the time-aware attention module (Figure A1(D)), although the trending of the timestamps appeared, most activities were skewed to the very beginning of the process, meaning that the time-intervals between activities were not well learned by the model. Concatenating the timestamp features did not give the transformer model enough information about the interactions between activities and the time-intervals. Without auxiliary activity frequency-based loss (Figure A1(A)), the model could not learn a gradual pattern of the timestamps, which indicated that the auxiliary losses were also needed for timestamp generation. The SeqGAN_T_ result (Figure A1(F)) also showed a clear trend because the timestamps of the SeqGAN_T_ sequences were sampled from the real timestamp distribution. Since the SeqGAN_T_ model generated sequences with shorter lengths, the timestamps for the back assessment (-BK) activities were not accurately align with the authentic data.

Based on the ProcessGAN timestamps, some activities (e.g., the -LUE or -RUE activities) were skewed to the end of the process. This scenario might arise due to the expansive exploration space of the ProcessGAN generator, making it challenging to converge to the exact same distribution as the authentic data. Consequently, some rarely observed data having more occurrences in the generated data. Although the ProcessGAN generated some timestamps that were different from the authentic processes, these timestamps were still valid according to the domain-expert knowledge.

##### Supervised Learning Score.

We used the false positive rate as the metric to measure the authenticity (Table 4). Because the transformer-based classification model can capture more intermediate dependencies between the tokens, a higher score suggested that the model managed generating sequences with complex activity dependencies and activity-timestamp relations. The more processes were classified as positive, the better our process generator performed.

The ProcessGAN model had better scores than other models for all datasets, showing that activities in the generated sequences had a more valid order and that timestamp of each activity was more accurate. The SeqGAN_T_ model had better results in activity type occurrence and sequence variance (Table 3) but did not always have higher authenticity scores (Table 4). Models that capturing global similarity between sequences did not always capture adequate local activity dependencies. ProcessGAN’s transformer-based generator outperformed the LSTM-based SeqGAN_T_ because the multi-head attention mechanism achieved better performance for longer sequences. Without the auxiliary loss values, the authenticity score dropped for all datasets, which is also observed in the statistical measurements (Table 3). For the SEP dataset, the ProcessGAN variants had good performance even without auxiliary losses and attention module, i.e., the ProcessGAN architecture was robust with simpler processes and sufficient data size.

##### Models Comparison.

We compared the runtime and the number of parameters between SeqGAN_T_, ProcessGAN and ProcessGAN without time-aware attention (i.e., GAN with two vanilla transformer models). The runtime was calculated based on one training iteration of the generator and discriminator, with a batch size of 64 (Table 5). The SeqGAN_T_ model had longer runtimes due to its reliance on a roll-out mechanism. As the sequence was generated through autoregression, the model simulated the entire sequence every time a token was generated to determine the loss value for that token. This roll-out mechanism increased the runtime of SeqGAN_T_. The parameter numbers of different models were comparable. The time-aware attention-based discriminator of ProcessGAN model had fewer parameters than the transformer-based discriminator but achieved better performance in discriminating sequences regarding the timestamps. These findings show that our model is efficient and effective.

#### 4.3.2 Case Study.

##### Workflow Discovery.

**Fig. 8.**
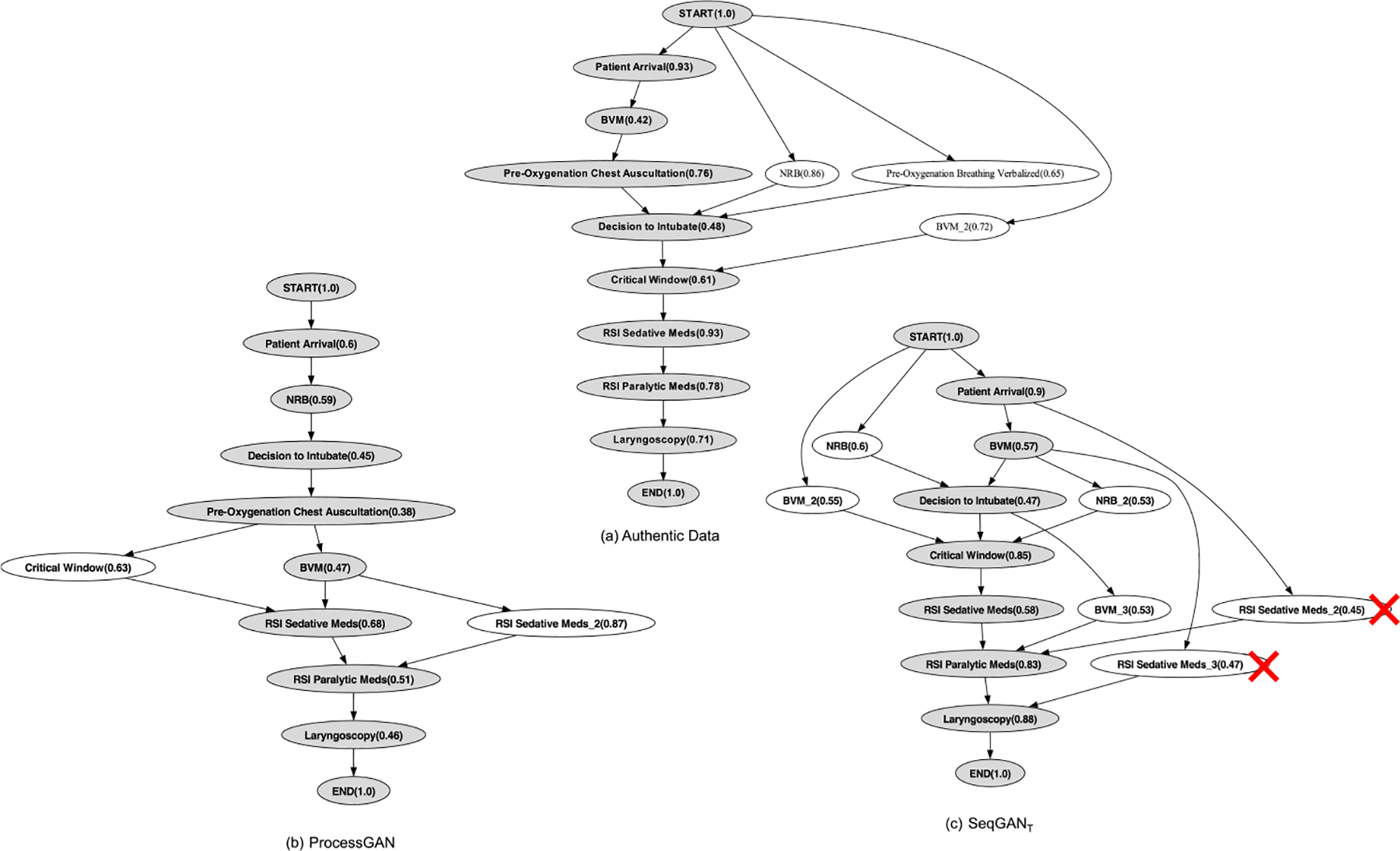
The workflow diagrams of the authentic INT dataset and two workflows discovered from synthetic data by the alignment method [5, 20]. The gray ovals are the backbone activities that represent the main process, and the white ovals are the side-branch activities that represent the parallel activities. The numbers in the brackets represent the frequency of each activity. The mismatches are marked with red signs (a cross indicated an incorrect order).

We used the INT dataset as a case study to discover the workflow diagrams and further evaluate the quality of the synthetic process data (Figure 8). We generated workflows for the authentic traces, the SeqGANT-generated traces and the ProcessGAN-generated traces. In synthetic workflows, the order of “patient arrival,” “**non-rebreather mask (NRB)**,” “**bag-valve mask (BVM)**,” “decision to intubate,” “critical window,” “**rapid sequence intubation (RSI)** sedative meds,” “RSI paralytic meds,” and “laryngoscopy” activities matched the authentic workflow.

Several discrepancies occurred between the synthetic and authentic workflows, including different activity orders and positions. To address these discrepancies, we asked a medical expert to evaluate the clinical applicability of the SeqGAN_T_ and ProcessGAN synthetic workflows:

- The activity “pre-oxygenation breathing verbalized” precedes “decision to intubate” in the authentic workflow. This activity followed the “decision to intubate” in the ProcessGAN workflow. Our medical expert confirmed that these activities could occur after the decision to intubate.
- Additional “RSI sedative meds” activities occurred between the “Patient Arrival” and the “Laryngoscopy” activity in the SeqGAN_T_ workflow. Our expert stated that “RSI sedative meds” must immediately precede “RSI paralytic meds” and cannot occur between the “Patient Arrival” and the “Laryngoscopy.”

The medical expert found that both synthetic workflows captured the major treatment steps of INT. As LSTM-based model did not capture every long-range dependency, SeqGAN_T_ tended to generate shorter traces. More BVM and NRB activities occurred in the processes, meaning that the temporal information was not well captured by the SeqGAN_T_ model. “Pre-oxygenation chest auscultation” was not observed in the SeqGAN_T_ model, meaning that the activity frequency generated by SeqGAN_T_ was inadequate.

The ProcessGAN traces provided more precise workflows for INT procedure. The medical expert favored the ProcessGAN workflow because it included longer traces and plausible pathways. The parallel pathways and intermediate dependencies were valid in the ProcessGAN synthetic workflow. Although the order of the activities was not exact the same as in the authentic workflow, it was still correct based on domain knowledge. ProcessGAN did not learn to “copy” the authentic data but learned to “imitate” the structural properties of the authentic data. It generated additional unobserved traces and augmented the process datasets.

It should be noted that in real-world medical settings, processes are flexible and variant because of different patient conditions and it is possible that the real process contains human errors. Even at major trauma centers with experienced teams, there were a certain number of preventable deaths that are related to errors during trauma resuscitation process [33]. Take trauma resuscitation for example, PM techniques can help medical providers identify which treatment activities are more likely to delay by analyzing the process data. By pinpointing these bottlenecks, interventions can be targeted to reduce the delays, such as ensuring faster availability of necessary equipment. Analyzing the errors is also an important topic in the medical field. Since ProcessGAN simulated the distribution of authentic data and can show the flexibility and variance that real data contains, the generated data could contain unobserved human errors that could happen in real process. Such process error analysis is important to medical process improvement and aiming for better patient outcomes.

##### Context Generation Results.

**Fig. 9.**
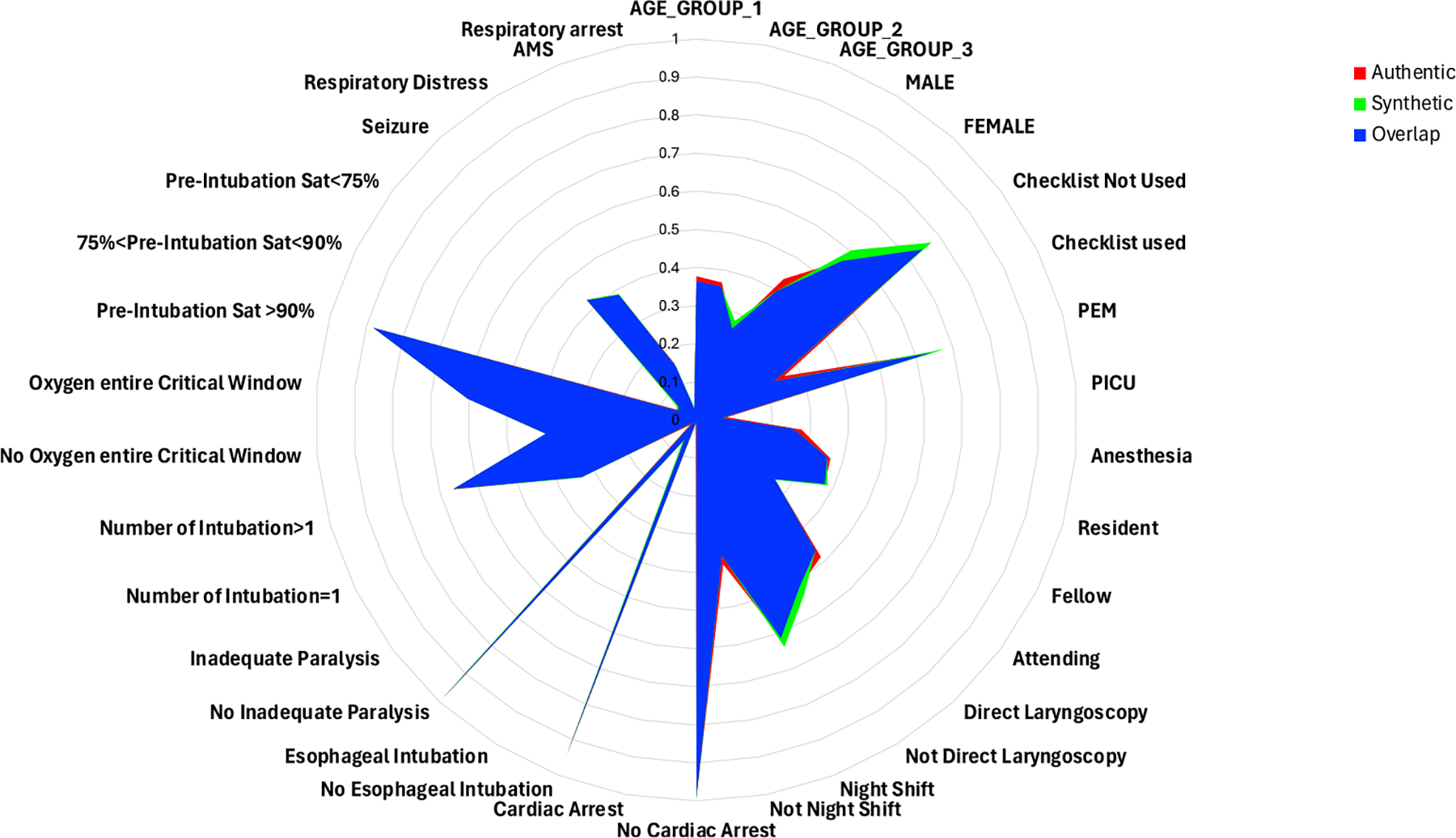
The distributions of the authentic patients’ context attributes, and the synthetic ones generated by our context generator (contexts attributes including: age, gender, checklist used by team leader or not, number of INTs, specialty of the team, training level of the team, direct laryngoscopy or not, night shift or not, reason, esophageal INT or not, inadequate paralysis or not, cardiac arrest or not, oxygenation entire critical window or not, pre-INT saturation category).

**Fig. A2.**
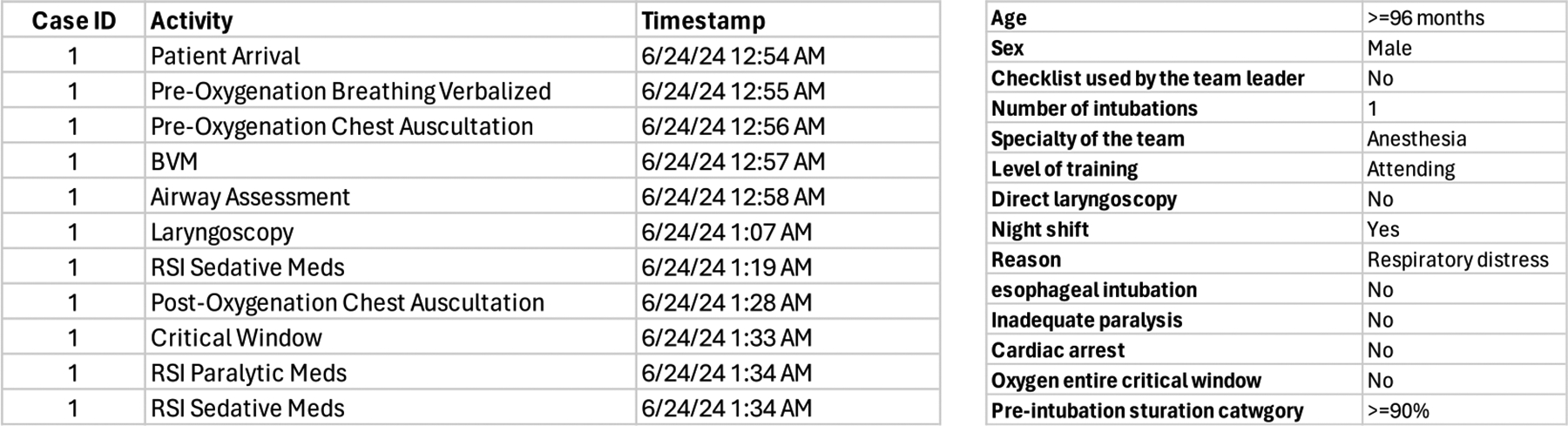
A generated example of the INT process with sequences (left) and patient contexts (right). The start time is set as the timestamp when we ran the ProcessGAN model, and the duration is randomly sampled based on the authentic process duration distribution.

**Fig. A3.**
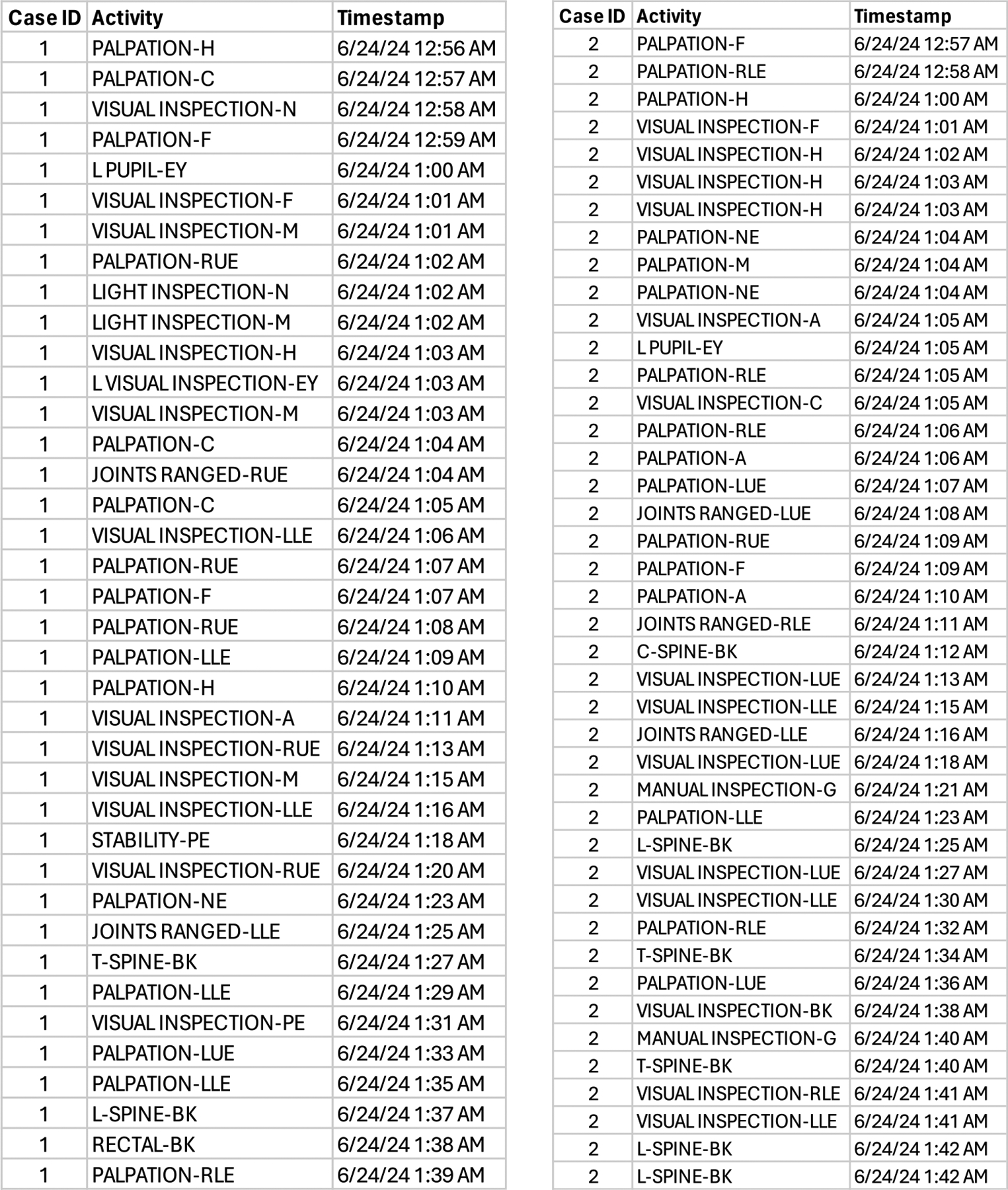
Two generated examples of the secondary survey process. The start time is set as the timestamp of running the ProcessGAN model, and the duration is set as random values sampled based on the authentic duration distribution. The contexts were not generated because we do not possess patient context for this dataset.

We performed a case study to evaluate context generation task on the INT dataset. The dataset contains 14 patient contexts, including team member characteristics, time of patient arrival, patient demographics, injury characteristics, and resuscitation characteristics. Due to the label imbalance, we used the weighted precision, recall, and F1-score to measure the performance of the context generator model (Table 6). The MLP-based single-task generator and the transformer-based multi-task context generator outperformed the random selection and ZeroR method on 8 of the 14 contexts. For the remaining 6 contexts, the ZeroR, MLP, and transformer methods had similar performances. The similar performance in these contexts can be explained because these contexts did not have strong correlations with the process traces. For example, the order of the INT activities may have trivial dependencies based on whether the team leader has used a checklist to log the patients’ conditions (the “checklist” category in Table 6). Some context labels were imbalanced in the dataset (e.g., esophageal INT, inadequate paralysis, cardiac arrest, and pre-INT saturation category, Table 6), which may have resulted in the good performances of the ZeroR method. With our trained context generator, we performed inference on the 101 synthetic INT process traces. Although our context generator has limitations in predicting precise contexts, it performed well in generating context data with the similar distribution to the authentic data at aggregated level (Figure 9). We aimed for the context generator to bridge the gap between process sequence distribution and context distribution, allowing us to leverage synthetic processes to generate synthetic latent contexts. In the Appendix section, Figures A2 and A3 show some of the generated process cases, including the sequences and the context attributes.

## 5 Limitation and Future Work

While our model generates timestamps for each activity, it currently does not account for the duration of individual activities. It’s important to note that different process datasets may vary in the levels of timestamp information. While some datasets only provide a single timestamp (usually the start time) of activities, others may include the end time or duration of each activity. In datasets where activity duration is available, it may be a crucial feature of the process. Specifically, in medical processes, the duration of some particular treatment activities may impact patient outcomes. Take trauma resuscitation for example, an injured patient may need ventilation to provide breathing support. The duration of ventilation is correlated to the probability of brain injury. In our future work, we intend to integrate duration information into our model, enabling the generation of both start and end timestamps for activities, thus enhancing the comprehensiveness of the process data.

It is important to recognize that generative models, such as GPT and DALL-E, can potentially produce misleading information due to various reasons including training data limitations, model overgeneralization, and so forth [11, 17]. There is a growing number of research in AI ethics and robustness aimed at creating models that are less likely to generate misleading information. ProcessGAN, as a generative model, shares the same limitation. Although addressing these concerns is out of scope of this article, we believe future research on synthetic process data generation should also consider these aspects.

## 6 Conclusion

Due to the privacy and confidentiality concerns, most process data cannot be shared publicly. Our research approached this problem by proposing a framework for generating synthetic process data that consists of activity and timestamp sequences. The framework works by first training generative models to learn the distribution of process data and uses the learned model to generate synthetic process data that can be shared publicly. We introduced ProcessGAN, a conditional GAN based on a transformer-based generator and a time-aware multi-head self-attention-based discriminator. ProcessGAN takes a predefined total duration as conditional input, and outputs the activity and the timestamp sequence that reflect this inputted duration. We further designed customized loss functions for the ProcessGAN model to better converge to the desired space. Our results showed that our ProcessGAN network could generate processes with parallel pathways and complex activity dependencies, and learn the relations between the activity, timestamp, and time intervals. In addition, we introduced a context generator that can generate multiple contexts associated with the processes. We proposed evaluation methods to assess the quality of the generated process data. Compared to other models, ProcessGAN can augment the existing process dataset even in the cases of complex process data with small-sized dataset and is efficient in the training process. Finally, the augmented process data reduces the effort of data collection and can be shared publicly.

## Figures and Tables

**Table 1.** Statistics of Three Medical Datasets and Two Public Datasets, Including the Number of Cases, Mean Value and Standard Deviation of the Sequence Length, Mean Value and Standard Deviation of the Total Process Duration, and the Number of Activity Types

	SS	INT	AIR	SEP	BPI
# Cases	271	101	53	900	3712
Length (Mean, Std.)	46.03±15.20	12.26±2.53	31.72±18.08	15.19±6.94	44.52±14.17
Duration (Mean, Std)	553.22±374.40(s)	1,234.26±760.84(s)	1,820.19±1080.05(s)	782.24±1537.87(h)	509.85±272.14(h)
# Act. Types	47	15	38	17	37

SS: The secondary survey of trauma resuscitation; INT: The intubation process of trauma resuscitation; AIR: The airway assessment of trauma resuscitation; SEP: Sepsis process; BPI: Loan application process dataset.

**Table 2.** The Configuration of the ProcessGAN Model for Each Dataset

	SS	INT	AIR	SEP	BPI
Batch size	64	32	32	256	512
Embedding	8	4	8	8	8
Hidden_g	64	64	64	64	64
Hidden_d	8	8	8	8	8
# head_g	4	4	4	4	4
# head_d	2	2	2	2	2
# layer_g	3	3	3	3	3
# layer_d	2	2	2	2	2
Dropout_g	0.1	0.1	0.1	0.1	0.1
Dropout_d	0.5	0.5	0.5	0.1	0.1
Learning rate_g	10^−3^	10^−3^	10^−4^	10^−4^	10^−4^
Learning rate_d	10^−3^	10^−3^	10^−4^	10^−4^	10^−4^

**Table 3.** The Statistical Measurements of Each Model for All Datasets

Dataset	Model	Length	Sequence Variance	Activity Type Occurrence Error	Activity Timestamps Error (mean)	Activity Timestamps Error (90% percentiles)
SS	Authentic Data	46.03±15.19	0.23	-	-	-
	SeqGAN_T_	31.40±17.02	0.29	0.11	10.52	19.58
	ProcessGAN	**45.96**±**8.77**	**0.23**	**0.06**	**4.82**	**5.71**
	w/o time loss	47.53±13.83	0.25	**0.06**	7.20	**7.16**
	w/o act loss	37.29±2.33	0.20	0.53	6.16	10.98
	w/o both loss	49.95±12.14	**0.24**	0.22	11.5	9.42
	w/o attention	**47.05±10.45**	**0.24**	0.12	**6.15**	10.47
	w/o attention and loss	65.48±7.49	0.17	1.04	11.26	21.21
INT	Authentic Data	12.26±2.53	0.15	-	-	-
	SeqGAN_T_	9.06±2.23	**0.18**	0.20	**1.21**	**2.46**
	ProcessGAN	**12.31±3.46**	**0.20**	**0.11**	**1.13**	**2.40**
	w/o time loss	12.98±5.22	0.25	0.13	2.70	3.80
	w/o act loss	**12.41±3.27**	0.22	0.46	1.82	2.56
	w/o both loss	9.50±4.18	**0.20**	0.83	3.62	4.08
	w/o attention	11.52±7.47	0.32	**0.11**	4.77	4.00
	w/o attention and loss	15.0±6.99	0.29	0.46	4.75	4.00
AIR	Authentic Data	31.71±18.08	0.26	-	-	-
	SeqGAN_T_	20.65±21.19	0.36	0.58	11.18	14.35
	ProcessGAN	**32.16±23.31**	**0.32**	**0.54**	**6.92**	**10.99**
	w/o time loss	**33.20±24.61**	**0.32**	**0.24**	12.20	12.67
	w/o act loss	39.33±28.92	0.33	0.65	**7.86**	**10.45**
	w/o both loss	29.90±21.68	**0.30**	0.62	14.80	12.40
	w/o attention	33.43±26.57	0.34	0.55	10.90	12.69
	w/o attention and loss	28.09±20.06	**0.32**	0.70	15.49	12.70
SEP	Authentic Data	15.19±6.94	0.17	-	-	-
	SeqGAN_T_	16.15±8.12	**0.20**	0.23	5.12	13.36
	ProcessGAN	**15.87±6.22**	**0.22**	**0.22**	**1.38**	**1.89**
	w/o time loss	16.15±10.78	**0.22**	**0.15**	4.39	5.93
	w/o act loss	**15.38±8.53**	0.23	0.29	**2.97**	**4.60**
	w/o both loss	24.5±7.65	0.14	1.32	4.24	6.32
	w/o attention	15.96±16.94	0.26	0.25	3.79	5.50
	w/o attention and loss	12.84±11.51	**0.22**	0.35	6.52	9.87
BPI	Authentic Data	44.52±14.17	0.18	-	-	-
	SeqGAN_T_	**40.32±12.84**	**0.17**	**0.25**	26.15	45.95
	ProcessGAN	**43.00±12.25**	**0.19**	0.40	**5.62**	**3.39**
	w/o time loss	50.64±28.23	0.22	0.88	**7.07**	9.38
	w/o act loss	44.57±32.54	0.29	1.09	11.18	9.23
	w/o both loss	41.95±25.26	0.25	0.59	9.92	7.46
	w/o attention	42.67±8.70	0.20	**0.29**	8.47	**7.00**
	w/o attention and loss	55.29±18.98	0.24	0.83	9.05	11.42

We marked the top 2 performances for each measurement in bold.

**Table 4.** The Fraction of the Synthetic Sequences That the “Off-the-Shelf” Binary Classifier Classified as Authentic

	SS	INT	AIR	SEP	BPI
F1 score	85.71	96.30	90.91	82.76	80.67
SeqGAN_T_	0.50	0.68	0.56	0.62	0.44
w/o time loss	0.77	0.79	0.65	0.97	0.47
w/o act loss	0.61	0.60	0.65	0.97	0.52
w/o both loss	0.54	0.56	0.59	0.94	0.47
w/o attention	0.74	0.77	0.65	0.97	0.63
w/o attention and loss	0.43	0.46	0.56	0.96	0.32
ProcessGAN	**0.81**	**0.84**	**0.72**	**0.99**	**0.74**

This fraction measures how frequently the synthetic data “tricked” the binary classifier. It is formally defined as the false positive rate (FPR=FP/N), i.e., the count of synthetic sequences misclassified as authentic, and N is the count of all negatives, i.e., synthetic sequences.

**Table 5.** The Comparison of Each Model, Including the Number of Parameters of Generator and Discriminator, and the Runtime of a Training Iteration (Batch Size = 64)

		SS	INT	AIR	SEP	BPI
SeqGANT	runtime (ms)	4,235	203	2,301	1,405	4,212
	# para G	7,385	2,089	7,016	7,282	6,934
	# para D	584	598	782	1,070	766
ProcessGAN	runtime (ms)	39	21	60	20	53
	# para G	7,873	4,465	4,764	4,478	4,738
	# para D	3,809	1,009	1,193	1,017	1,177
W/O attention	runtime (ms)	31	24	45	22	35
	# para G	7,873	7,201	4,764	4,478	4,738
	# para D	9,409	8,321	2,553	2,025	2,841

**Table 6.** The Performance of Random Selection, ZeroR, MLP-Based Single-Task Context Generator, and Transformer-Based Multi-Task Context Generator on Different Patients’ Context Attributes

Contexts Category	random			ZeroR			MLP-based Context Generator			Transformer-based Context Generator		
	precis ion	recall	F1-score	precis ion	recall	F1-score	precis ion	recall	F1-score	precis ion	recall	F1-score
Age category	0.33	0.32	0.32	0.14	0.38	0.21	0.39	0.35	0.35	0.43	0.35	0.35
Sex	0.49	0.49	0.49	0.32	0.56	0.41	0.44	0.43	0.43	0.53	0.51	0.51
Checklist	0.58	0.45	0.48	0.55	0.74	0.63	0.63	0.62	0.62	0.61	0.59	0.60
INT category	0.51	0.46	0.47	0.44	0.66	0.53	0.61	0.59	0.59	0.63	0.57	0.58
Specialty	0.50	0.33	0.39	0.41	0.64	0.50	0.42	0.42	0.42	0.44	0.46	0.45
Level of training	0.32	0.32	0.32	0.14	0.38	0.21	0.40	0.39	0.38	0.33	0.30	0.29
Direct laryngoscopy	0.49	0.49	0.49	0.27	0.51	0.35	0.50	0.48	0.48	0.54	0.51	0.51
Night shift	0.51	0.48	0.48	0.38	0.61	0.47	0.45	0.44	0.43	0.68	0.65	0.65
Reason	0.26	0.18	0.20	0.18	0.43	0.25	0.37	0.33	0.33	0.33	0.34	0.32
Esophageal INT	0.90	0.59	0.70	0.88	0.94	0.91	0.89	0.87	0.87	0.93	0.89	0.90
Inadequate paralysis	0.96	0.52	0.67	0.96	0.98	0.97	0.96	0.97	0.96	0.96	0.96	0.96
Cardiac arrest	0.99	0.50	0.66	0.98	0.99	0.99	0.98	0.99	0.98	0.98	0.99	0.99
Oxygenation entire critical window	0.54	0.50	0.50	0.36	0.60	0.45	0.54	0.54	0.53	0.46	0.45	0.44
Pre-INT saturation category	0.84	0.39	0.49	0.78	0.88	0.83	0.77	0.77	0.77	0.80	0.82	0.81
mean	0.58	0.42	0.47	0.47	0.66	0.54	0.60	0.59	0.58	0.62	0.60	0.60

The context categories including: age (1 < 24 months; 2 = 24 to 96 months; 3 > 96 months), sex (1 = Male, 2 = Female), checklist used by team leader (1 = no, 2 = yes), number of intubations (1 = 1, 2 > 1), specialty of the team (1 = PEM, 2 = PICU, 3 = Anesthesia), training level of the team (1 = resident, 2 = fellow, 3 = attending), direct laryngoscopy (1 = no, 2 = yes), night shift (1 = night, 2 = not night), reason (1 = seizure, 2 = respiratory distress, 3 = AMS, 4 = respiratory arrest), esophageal intubation (1 = no, 2 = yes), inadequate paralysis (1 = no, 2 = yes), cardiac arrest (1 = no, 2 = yes), oxygenation entire critical window (1 = no, 2 = yes), pre-intubation saturation category (1 > or = 90%, 2 = 75%–89%, 3 < 75%).

## Box

CCS Concepts: • **Computing methodologies** → **Artificial intelligence;** • **Information systems** → **Data mining;**
